# Women's empowerment, production choices, and crop diversity in Burkina Faso, India, Malawi, and Tanzania: a secondary analysis of cross-sectional data

**DOI:** 10.1016/S2542-5196(23)00125-0

**Published:** 2023-07-10

**Authors:** Kaela Connors, Lindsay M Jaacks, Ananya Awasthi, Karoline Becker, Rachel Bezner Kerr, Emily Fivian, Aulo Gelli, Helen Harris-Fry, Jessica Heckert, Suneetha Kadiyala, Elena Martinez, Marianne V Santoso, Sera L Young, Lilia Bliznashka

**Affiliations:** a Harvard T H Chan School of Public Health, Boston, MA, USA; b Global Academy of Agriculture and Food Systems, University of Edinburgh, Midlothian, UK; c Anuvaad Solutions, New Delhi, India; d Department of International Development, University of Oxford, Oxford, UK; e Department of Global Development, Cornell University, Ithaca, NY, USA; f Department of Population Health, London School of Hygiene & Tropical Medicine, London, UK; g International Food Policy Research Institute, Washington, DC, USA; h Friedman School of Nutrition Science and Policy, Tufts University, Boston, MA, USA; i Department of Anthropology, Northwestern University, Evanston, IL, USA

## Abstract

**Background:**

Bolstering farm-level crop diversity is one strategy to strengthen food system resilience and achieve global food security. Women who live in rural areas play an essential role in food production; therefore, we aimed to assess the associations between women's empowerment and crop diversity.

**Methods:**

In this secondary analysis of cross-sectional data, we used data from four cluster-randomised controlled trials done in Burkina Faso, India, Malawi, and Tanzania. We assessed women's empowerment using indicators from the Women's Empowerment in Agriculture Index. Farm-level crop diversity measures were the number of food crops grown, number of food groups grown, and if nutrient-dense crops were grown. We used a two-stage modelling approach. First, we analysed covariate-adjusted country-specific associations between women's empowerment and crop diversity indicators using multivariable generalised linear models. Second, we pooled country-specific associations using random-effects models.

**Findings:**

The final analytic sample included 1735 women from Burkina Faso, 4450 women from India, 547 women from Malawi, and 574 women from Tanzania. Across all countries, compared with households in which women provided input into fewer productive decisions, households of women with greater input into productive decisions produced more food crops (mean difference 0·36 [95% CI 0·16–0·55]), a higher number of food groups (mean difference 0·16 [0·06–0·25]), and more nutrient-dense crops (percentage point difference 3 [95% CI 3–4]). Across all countries, each additional community group a woman actively participated in was associated with cultivating a higher number of food crops (mean difference 0·20 [0·04–0·35]) and a higher number of food groups (mean difference 0·11 [0·03–0·18]), but not more nutrient-dense crops. In pooled associations from Burkina Faso and India, asset ownership was associated with cultivating a higher number of food crops (mean difference 0·08 [0·04–0·12]) and a higher number of food groups (mean difference 0·05 [0·04–0·07]), but not more nutrient-dense crops.

**Interpretation:**

Greater women's empowerment was associated with higher farm-level crop diversity among low-income agricultural households, suggesting that it could help enhance efforts to strengthen food system resilience.

**Funding:**

Bill & Melinda Gates Foundation.

## Introduction

The stability of global food systems and their ability to nourish the global population are threatened by crossing planetary boundaries.^1^ Greater crop diversity at the national level can contribute to food security by stabilising food production.^2^ However, crop diversity at different geospatial scales (eg, plot-level, farm-level, landscape-level, and national-level) is interdependent (ie, diversity at larger scales is dependent on diversity at smaller scales and vice versa) and dynamic. The benefits of crop diversity—in terms of food security, farmers’ income, and environmental health—depend on the spatial scale.^3^ Overall, 80% of farmers in the world are smallholders,^4^ thus on-farm food production requires attention to ensure the wellbeing of households that predominantly rely on agriculture for both food and income and to maximise co-benefits to planetary health.

The conventional narrative of agricultural development is that increased farm-level specialisation, rather than diversity, is needed to increase participation in markets.^5^ However, in the past two decades, an alternative narrative has emerged emphasising the value of farm-level diversification to improve resilience to market variability, weather variability, extreme weather events, and pests and disease.^6^ Several analyses in low-income and middle-income countries (LMICs), where most farms are small (<2 hectares),^4^ have demonstrated that agricultural households, particularly low-income ones,^7^ that grow a greater diversity of crops, have higher and more stable agricultural incomes and are less likely to be low-income.^8^, ^9^, ^10^, ^11^ A 2020 systematic review of 98 meta-reviews and more than 6000 studies found that agricultural diversification at multiple scales and across contexts supports yields, in addition to many ecosystem services, including enhanced soil fertility, buffering farms against climate change.^12^

**Research in context**

**Evidence before this study**

We searched PubMed for quantitative studies, systematic reviews, randomised studies, and quasi-randomised studies published between Jan 1, 2017, and March 5, 2022, using the search terms “women* empowerment” and “crop diversity” or “production diversity”. Our search yielded 35 results. We identified two studies in low-income and middle-income countries (LMICs) that evaluated the association between women's empowerment and crop diversity in India and Bangladesh. The findings of both studies indicated that women who participated in self-help groups, which are hypothesised to foster women's empowerment, grew more diverse crops and were more likely to grow food crops. The study in Bangladesh also found that women who were more empowered in certain domains, specifically higher input in decision making and active membership in community groups, were more likely to allocate land to nutrient-dense crops, such as fruits and vegetables. However, it was unclear whether these observed associations held across contexts (ie, were generalisable to other contexts) and whether other domains of women's empowerment were associated with greater crop diversity, which is important for food systems resilience.

**Added value of this study**

We examined the association between women's empowerment and farm-level crop diversity using cross-sectional data from four studies in LMICs: Burkina Faso, India, Malawi, and Tanzania. We disaggregated women's empowerment into five domains, which allowed us to parse out the aspects of empowerment associated with crop diversification. We used measures of crop diversity that reflect cultivation of diverse and nutritious crops. We found that the associations between women's empowerment and crop diversity varied by domain of women's empowerment and by context. Our analysis is unique in that we investigated this association across four different contexts and two global regions, providing insight into associations within and between contexts. Additionally, our analysis investigated specific domains of women's empowerment and their association with three different measures of crop diversity to enable more targeted policy translation.

**Implications of all the available evidence**

Taken together, the evidence indicates that women's empowerment is positively associated with crop diversity, and that the strength of this association varies by context and the indicators of women's empowerment and crop diversity examined. Increased input into decision making, asset ownership, and participation in community groups might be especially important for diversification of cropping systems. Efforts to strengthen food system resilience could consider women's empowerment as a pathway to benefit agricultural production diversity, recognising that the links between women's empowerment and agricultural production are complex and context dependent. Although women's empowerment is imperative from a human rights perspective and as a normative goal, it might also reinforce other agendas, such as the provision of healthy diets that support a healthy planet.

Cultivating a greater diversity of crops can also improve diets directly through increasing food availability at home, or indirectly, through improving incomes from crops sold to market and the ability to purchase nutritious foods.^13^ However, evidence suggests that this effect is small in magnitude and context-specific.^14^ The effect is stronger, although small, in subsistence-oriented contexts versus market-oriented contexts and in sub-Saharan Africa versus other regions.^14^

In contexts where farm-level crop diversification is desirable (ie, smallholder farms in LMICs with poor market access), interventions that empower women involved in agriculture might be one pathway to achieving this outcome.^15^, ^16^, ^17^ Empowerment of women, whereby women have the resources, ability, and agency to exercise choice as per Kabeer's definition,^18^ is high on the global development agenda: the contribution of women in smallholder agricultural production has been highlighted in the Sustainable Development Goals. Although a complex and multidimensional construct, women's empowerment might improve women's agency within the (often multigenerational) household and outside the household, thus enabling greater access to resources and services. Subsequently, the empowerment of women in agriculture might positively impact farm-level crop diversity. Women currently have low influence on agriculture in some locations because they have traditionally had less control over agricultural decisions,^19^ less secure ownership of land and limited access to agricultural inputs,^19^ and fewer opportunities to receive training and entitlements.^19^ Men and women might also have different priorities.^20^, ^21^ Context-dependent evidence shows that women who are typically more responsible for nourishing their families focus on food cropping, whereas men who are often more responsible for providing income for the family invest more in cash cropping.^22^ Thus, it is plausible that if women are empowered, they will more actively participate in agricultural decision making and might allocate land, labour, and other productive inputs differently from men and other household members, with more focus on producing a greater diversity of crops, including more nutrient-rich crops (appendix pp 5–6).

To the best of our knowledge, only two studies have evaluated the association between women's empowerment and crop diversity, showing that more empowered women are more likely to cultivate more crops and more nutrient-dense crops than less empowered women in India and Bangladesh.^23^, ^24^ However, one study only investigated women's participation in self-help groups and the other assessed women's empowerment in agriculture in relation to crop land allocation not crop diversity. Another study in Nepal investigated interactions between women's empowerment in agriculture and crop diversity on women's and children's diet and nutritional status and showed that women's empowerment influenced the negative association between low crop diversity and children's diet and nutritional status.^25^ Our understanding of these associations in other populations in south Asia and in other regions is limited. There is a need to build on existing evidence by evaluating specifically which domains of women's empowerment are associated with crop diversity.

We aimed to investigate the association between the empowerment of women and farm-level crop diversity among low-income agricultural households using cross-sectional data from Burkina Faso, India, Malawi, and Tanzania. We hypothesised that greater empowerment of women would be associated with increased farm-level food crop diversity, food group diversity, and greater cultivation of nutrient-dense crops.

## Methods

### Study design and population

For this secondary analysis of cross-sectional data, we identified eligible studies on the basis of peer-reviewed articles that reported on women's empowerment, using the Women's Empowerment in Agriculture Index (WEAI),^26^ and agricultural production. Investigators were contacted to contribute to the analysis. The WEAI was selected because it was specifically designed to measure women's empowerment in agriculture. Detailed information and a comparison of each study site, including baseline information on ethnicity (available for India and Tanzania), is in the appendix (pp 9–19).

The data from Burkina Faso were obtained from SELEVER, a cluster-randomised controlled trial evaluating the effect of a poultry value chain intervention on women's and children's diets, health, and nutritional status in western Burkina Faso.^27^ In March, 2016, the 2016 baseline survey collected data from 1800 households in 120 villages in three regions. Households were included if they had a woman aged 15–35 years with at least one child aged 2–4 years living in the same household.^27^ In polygynous households, the wife who was most knowledgeable about poultry farming was interviewed.

Information from India comprised baseline data from the UPAVAN trial, a cluster-randomised controlled trial conducted in Odisha, India from 2016 to 2020.^28^ The trial tested the effect of women's group interventions involving nutrition-sensitive agriculture videos on maternal and child nutritional outcomes. The 2016–17 baseline survey (conducted between Nov 24, 2016, and Jan 24, 2017) included 4480 women from 148 villages in Keonjhar district. Participants were women aged 15–49 years with a child aged 0–23 months and their spouses. Women with disabilities and children with disabilities, or household members who had been residents for less than 6 months in the previous year, were excluded.

For Malawi, we used baseline data from a cluster-randomised controlled trial assessing the effectiveness of combining agriculture and nutrition activities with an early childhood development curriculum.^29^ The trial enrolled 1256 households from 60 communities in the Zomba district of southern Malawi between Sept 2, 2015, and Dec 4, 2015.^29^ Children aged 36–72 months and their caregivers living in communities with community-based childcare serviced by Save the Children's Early Childhood Health and Development were targeted.

Data from Tanzania were obtained from a cluster-randomised controlled trial assessing the impact of a nutrition-sensitive agroecological intervention on women and children's nutritional outcomes.^29^ We used baseline data from January, 2016, on 591 households from 20 villages in Singida rural district. Households were eligible if: they (1) were food insecure, (2) had a child younger than 1 year, (3) planned to engage in farming for the next year and had access to land, (4) planned to continue living in the village for the next 3 years, and (5) were interested in implementing new farming methods.

This study was exempt from full review by the Harvard T H Chan School of Public Health Institutional Review Board (IRB22–0339) because it included de-identified secondary data. Each trial received ethical approval from the relevant institutions in their respective countries. In each trial, informed consent was obtained from participants. Further details are published elsewhere.^27^, ^28^, ^29^, ^30^

### Exposure and outcome measures

All studies assessed women's empowerment using the WEAI, a standardised instrument that allowed data to be pooled across contexts. Full details of the WEAI have been outlined elsewhere^26^ and are summarised in the appendix (pp 6–7). Briefly, the WEAI has five domains of empowerment: (1) input into productive decisions, (2) access to and control over productive resources, referred to herein as asset ownership, (3) control over use of income, (4) leadership in the community, referred to herein as group membership, and (5) time allocation.^26^

We defined empowerment indicators using counts of achievements made in each area (table).^31^, ^32^, ^33^ Harmonisation of individual items and modification of binary indicators across studies are summarised in the appendix (pp 7, 11–12). As a sensitivity analysis, we calculated binary indicators categorising women as empowered or disempowered within a domain to assess the robustness of our results and facilitate interpretability (table).^32^ In additional sensitivity analyses, we used a version of the work balance indicator in which productive work hours added half the time spent on childcare, including when childcare was a secondary activity.^33^ Although the WEAI can be aggregated into total scores, we had incomplete information to compute them and focused our analysis on the individual indicators (appendix pp 11–12).

**Table:** Women's empowerment indicators*

	**Continuous version**†	**Binary version**‡	**Countries with available data**
Input into productive decisions	Number of domains an individual has some input into decisions or feels that they can make decisions or made decision themselves	Adequate if has some input or input into most or all decisions or made decision in two or more areas†	Burkina Faso, India, Malawi, Tanzania
Ownership of assets§	Number of agricultural assets owned (solely or jointly) by an individual; number of assets owned (solely or jointly) by an individual	Adequate if individual owns (sole or jointly) at least one large asset or at least two small assets	Burkina Faso, India
Access to and decisions about credit	Number of sources of credit that anyone in respondent's household (including respondent) borrowed or used in the previous 12 months; number of sources of credit that the individual solely made decision to either borrow from or how to use in the previous 12 months given that their household has access to that source of credit	Adequate if using at least one source of credit or made decision solely regarding at least one source of credit given that household has access to credit	Burkina Faso, India, Malawi, Tanzania
Group membership	Number of groups an individual is an active member of; if the individual reports no groups in their community, they are considered a member of 0 groups	Adequate if an active member in at least one group	Burkina Faso, India, Malawi, Tanzania
Workload	Number of hours dedicated to productive work per day by the respondent	Adequate if works <10·5 hours in previous day; adequate if works less than 10·5 hours in previous day, including childcare as a secondary activity¶	Burkina Faso, India, Malawi‖, Tanzania

A-WEAI=Abbreviated-Women's Empowerment in Agriculture Index. pro-WEAI=project-level Women's Empowerment in Agriculture Index.

* Specific items varied according to study (appendix pp 11–12).

† All indicators are based on those established for the A-WEAI. The pro-WEAI indicator for workload inspired the inclusion of childcare as a secondary activity;^31^ specific cutoffs and definitions were adjusted to accommodate data in this study (appendix p 7).

‡ Binary definitions obtained from the A-WEAI Instructional Guide by Malapit and colleagues.^32^

§ Small assets were defined as small livestock, non-mechanised farm equipment, or low-cost durables; large assets were defined as large livestock, agricultural land ownership, mechanised farm equipment, non-farm commercial equipment, a house or building, high-cost durables, mobile phone, non-agricultural land, or method of transport; a full list of assets (including agricultural and non-agricultural) per country is included in the appendix (p 15).

¶ Workload definition based on the pro-WEAI indicator definition.^33^

‖ Time dedicated to childcare was not available for Malawi.

We used total food crop count as a primary measure of crop diversity. Non-food cash crops (eg, cotton, tobacco, and spices) were excluded because they were not collected in all studies.

We also calculated two nutritionally important crop diversity measures to measure access to a diverse diet, which is important for diet quality. First, we calculated crop diversity measured as a count out of seven crop-related food group categories:^34^ (1) grains, roots, and tubers, (2) pulses, (3) nuts and seeds, (4) dark green leafy vegetables, (5) vitamin-A rich fruits and vegetables, (6) other vegetables, and (7) other fruit. Crop classifications are described in the appendix (pp 13–14). Second, to facilitate interpretation, we defined a binary indicator for whether the household cultivated nutrient-dense crops, in which nutrient-dense crops were defined as pulses, nuts and seeds, dark green leafy vegetables, vitamin-A rich fruits and vegetables, other vegetables, or other fruit.

### Statistical analysis

Although data were collected for cropping patterns in the previous 12 months in all studies, we focused on the primary growing season for each country: September to January for Burkina Faso, June to November for India, September to November for Malawi, and January to February for Tanzania. The final analytic sample size was determined on the basis of complete information on cropping patterns and women's empowerment. Data were excluded if information on cropping patterns or women's empowerment were missing.

We summarised sociodemographic, women's empowerment, and crop diversity variables by country. We evaluated the country-specific associations between indicators of women's empowerment and crop diversity using univariable and multivariable generalised linear models. We calculated covariate-adjusted mean differences and 95% CIs for total crop count and food group count and covariate-adjusted risk differences, reported as percentage point difference, for nutrient-dense crop production. We adjusted for covariates when information was available and when relevant by context. For example, Burkina Faso has the highest prevalence of polygyny in West Africa (around 36% of households), whereas the prevalence of polygyny is either low or non-existent in the other countries.^35^ Adjusted models controlled for intervention assignment, woman's age, education, asset score (appendix p 15), and household size. The Burkina Faso model additionally controlled for polygyny; the India model for owned land size; and the Tanzania model for cultivated land size. Missing values on any of the covariates were imputed using mean imputation (<5% for all variables across settings). The models for the asset ownership domain of women's empowerment did not control for asset score. Additionally, models for access to and decisions about credit also controlled for whether the woman was an active member of a credit or microfinance lending group. Since all data came from cluster-randomised controlled trials, SEs were clustered at the study-specific unit of randomisation, which was at the village or sub-village level.

In sensitivity analyses, we re-estimated the country-specific models using multivariable log-Poisson models to calculate relative risk. With a common binary outcome (the minimum and maximum ranges of households that produced nutrient-dense crops across studies was 947 [21·3%] of 4450 households in India and 504 [92·1%] of 547 households in Malawi, respectively), log-Poisson models overestimate SEs, producing wider confidence intervals and less precision than is true.^36^

We pooled multivariable country-specific estimates using a random-effects model using the DerSimonian and Laird method.^37^ Between-study heterogeneity was examined using the *I*^2^ test statistic and corresponding p values. Data management and statistical analyses were done using R (version 4.1).

### Role of the funding source

The funder of the study had no role in study design, data collection, data analysis, data interpretation, or writing of the report.

## Results

After the exclusion of women for whom complete data regarding cropping patterns and women's empowerment were not available, the final analytic sample included 1735 women from Burkina Faso, 4450 women from India, 547 women from Malawi, and 574 women from Tanzania (appendix p 16).

Women generally had similar sociodemographic characteristics across countries. Across all studies, mean age was 27·6 years (SD 7·6), most women were married, and most women had at least some education (appendix p 17).

Women's empowerment varied across settings (appendix pp 18–20). Women in Burkina Faso were most empowered with regard to work balance (1507 [86·9%] of 1735 women) and asset ownership (1596 [92·0%] women), whereas women in India were most empowered with regard to input into productive decisions (3691 [82·9%] of 4450 women) and asset ownership (4062 [91·3%] women). In Tanzania, women were most empowered with regard to input into productive decisions (540 [94·1%] of 574 women), whereas in Malawi they were most empowered with regard to input into productive decisions (355 [64·9%] of 547 women), access to and decisions on credit (353 [64·5%] women), and group membership (361 [66·0%] women).

Most households across settings cultivated grains (appendix p 21). Total food crop diversity and food group crop diversity varied across settings (appendix p 21). In India, most households cultivated one food crop, growing a mean of 1·36 crops (SD 1·43), whereas Malawi had the highest mean total food crop count (5·7 [3·37]) and mean food group count (3·94 [1·73]). Differences were observed in the cultivation of other food groups. Households that cultivated multiple crops in Burkina Faso and Malawi tended to grow pulses and dark green leafy vegetables, whereas households that cultivated multiple food crops in India tended to grow pulses and other vegetables and in Tanzania, other vegetables and nuts and seeds. Production of vitamin A-rich fruits and vegetables and other fruit was observed mainly in Malawi. Nutrient-dense crops were cultivated by 1436 (82·8%) of 1735 households in Burkina Faso, 947 (21·3%) of 4450 households in India, 504 (92·1%) of 547 households in Malawi, and 297 (51·9%) of 574 households in Tanzania.

**Figure 1:**
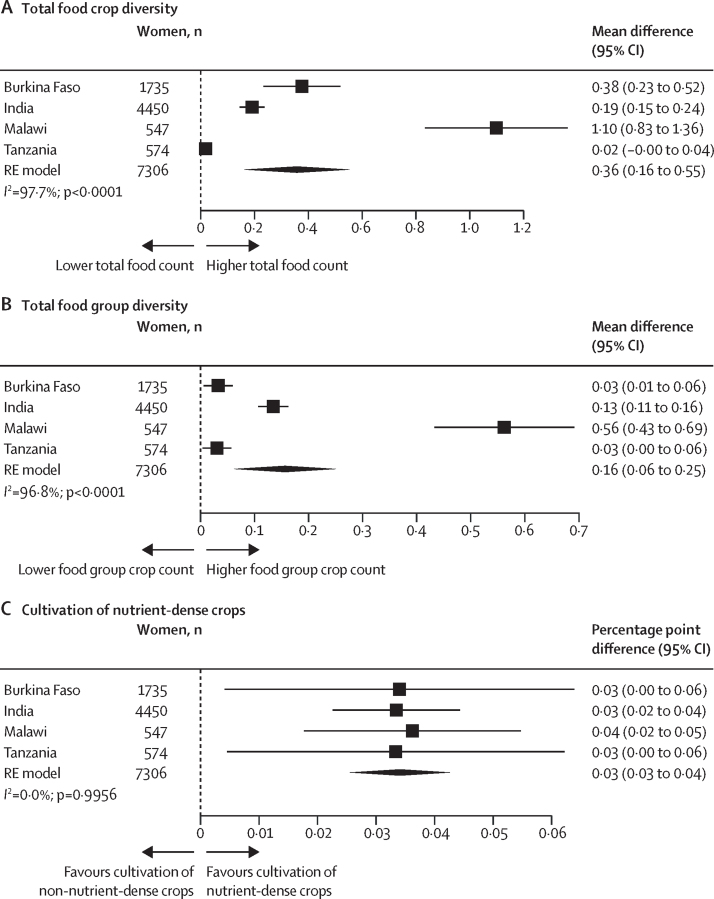
Association between women's input into productive decisions and total food crop diversity (A), total food group diversity (B), and cultivation of nutrient-dense crops (C) Total food group diversity ranged from zero to seven food groups. Cultivation of nutrient-dense crops was defined as the cultivation of at least one crop in the following food group categories: pulses, nuts and seeds, dark green leafy vegetables, other vitamin-A rich fruits and vegetables, other vegetables, or other fruit. Non-cultivators of nutrient-dense crops were farmers who grew only grains, roots, and tubers, or only grew non-food crops (ie, cash crops). Input into productive decisions was defined as the number of areas a woman had some input in decisions or felt she could make decisions or made decisions herself. Country-specific adjusted estimates were pooled using an RE model using the DerSimonian and Laird method. The adjusted model for Burkina Faso controlled for intervention assignment, age, educational attainment, asset score, household size, and polygyny. The model for India controlled for intervention assignment, age, educational attainment, asset score, household size, and land size. The model for Malawi controlled for intervention assignment, age, educational attainment, asset score, and household size. The model for Tanzania controlled for intervention assignment, age, educational attainment, asset score, household size, and land size. RE=random-effects.

Our pooled results indicated that specific domains of women's empowerment were differentially associated with farm-level crop diversity. Compared with households in which women had less input into productive decisions, households of women with greater input into productive decisions cultivated more food crops (mean difference 0·36 [95% CI 0·16–0·55]) and food group crops (0·16 [0·06–0·25]) and were more likely to produce nutrient-dense crops (percentage point difference 3 [95% CI 3–4]; figure 1). There was high heterogeneity in food crop (*I*^2^=97·7%) and food group (*I*^2^=96·8%) estimates across countries. For Tanzania, the continuous indicator of input into productive decisions was not associated with any measure of crop diversity; however, the binary indicator was associated with all three measures (appendix p 22).

**Figure 2:**
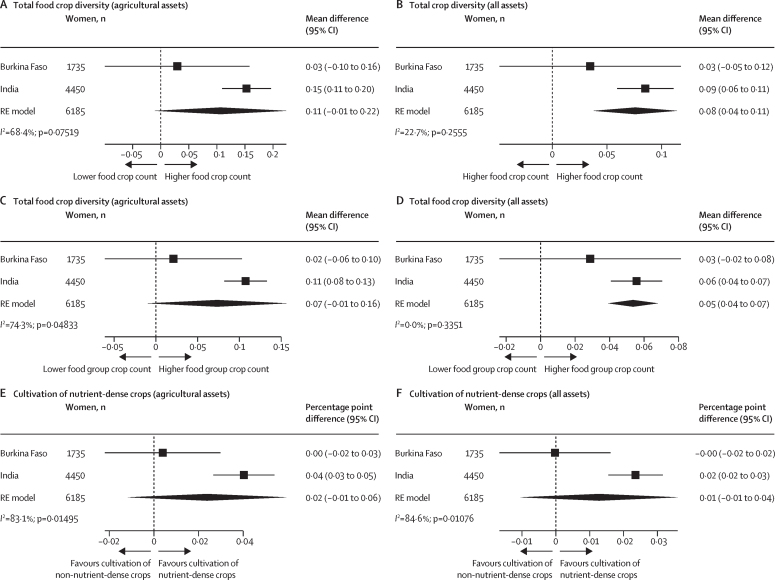
Association between ownership of assets (overall and agricultural only) and total food crop diversity, total food group diversity, and cultivation of nutrient-dense crops Association of agricultural (A) and all (B) assets with total food crop diversity. Association of agricultural (C) and all (D) assets with total food group diversity. Association of agricultural (E) and all (F) assets with cultivation of nutrient-dense crops. Total food group diversity ranged from zero to seven food groups. Agricultural assets were defined as the number of agricultural assets that the woman owned herself or jointly. All assets were defined as the number of assets (including agricultural assets) that the woman owned herself or jointly. Cultivation of nutrient-dense crops was defined as cultivation of at least one crop in the following food group categories: pulses, nuts and seeds, dark green leafy vegetables, other vitamin-A rich fruits and vegetables, other vegetables, or other fruit. Non-cultivators of nutrient-dense crops were those who grew only grains, roots, and tubers or if they grew no food groups (ie, not growing anything or only growing non-food crops [ie, cash crops]). We pooled country-specific adjusted estimates using an RE model using the DerSimonian and Laird method. The adjusted model for Burkina Faso controlled for intervention assignment, age, educational attainment, household size, and polygyny. The model for India controlled for intervention assignment, age, educational attainment, household size, and land size. RE=random-effects.

Data on women's ownership of agricultural assets were only available for Burkina Faso and India. On the basis of pooled associations, ownership of agricultural assets was associated with greater crop diversity (figure 2); however, associations were only significant in India (food crop count mean difference 0·15 [95% CI 0·11–0·20]; food group count 0·11 [0·08–0·13]; and nutrient-dense percentage point difference 0·04 [0·03–0·15]). Owning assets, in addition to agricultural assets, was significantly associated with all three crop diversity measures in India (food crop count mean difference 0·09 [95% CI 0·06–0·11]; food group count 0.06 [0·04–0·07]; and nutrient-dense risk difference 0·02 [0·02–0·02]). In sensitivity analyses, results using the binary indicator were consistent (appendix p 23).

**Figure 3:**
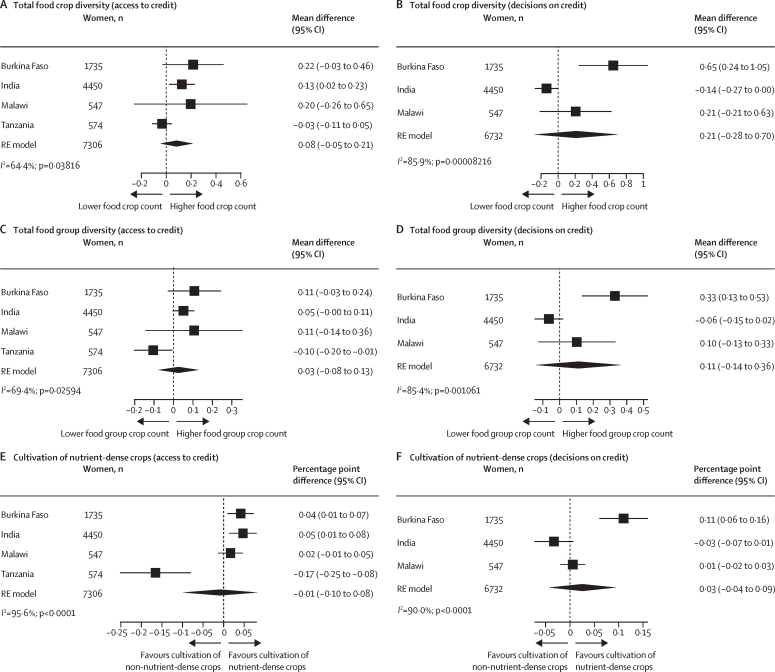
Association between access to, and decisions on, credit and total food crop diversity, total food group diversity, and cultivation of nutrient-dense crops Associations of credit use (A) and decisions made on credit use (B) and total food crop diversity. Associations of credit use (C) and decisions made on credit use (D) and total food group diversity. Associations of credit use (E) and decisions made on credit use (F) and cultivation of nutrient-dense crops. Credit use was defined as the number of sources of credit that anyone in the woman's household (including herself) has borrowed from or used in the past 12 months. Decisions on credit were defined as the number of sources of credit that the woman solely made the decision to either borrow from or how to use the credit in the past 12 months considering that their household has access to such sources of credit. Cultivation of nutrient-dense crops was defined as cultivating at least one crop in the following food group categories: pulses, nuts and seeds, dark green leafy vegetables, other vitamin-A rich fruits and vegetables, other vegetables, or other fruit. Non-cultivators of nutrient-dense crops were those who grew only grains, roots, and tubers or if they grew no food groups (ie, not growing anything or only growing non-food crops [ie, cash crops]). Country-specific adjusted estimates were pooled using an RE model using the DerSimonian and Laird method. The adjusted model for Burkina Faso controlled for intervention assignment, age, educational attainment, asset score, household size, polygyny, and active membership of a credit or microfinance lending group. The model for India controlled for intervention assignment, age, educational attainment, asset score, household size, land size, and active membership of a credit or microfinance lending group. The model for Malawi controlled for intervention assignment, age, educational attainment, asset score, household size, and active membership of a credit or microfinance lending group. The model for Tanzania controlled for intervention assignment, age, educational attainment, asset score, household size, land size, and active membership of a credit or microfinance lending group. RE=random-effects.

In pooled estimates, women's access to credit was not associated with crop diversity, but heterogeneity was high (*I*^2^ >64% for all crop diversity measures; figure 3). In India, each additional credit source that women had access to was positively associated with number of food crops grown [mean difference 0·13 [95% CI 0·02–0·23]) and nutrient-dense crop cultivation (percentage point difference 5 [95% CI 1–8]). In Tanzania access to credit was negatively associated with food group count (mean difference –0·10 [–0·20 to –0·01)] and nutrient-dense crop cultivation (percentage point difference –17 [–25 to –8]). No overall association was observed in the pooled estimates with regard to women's decisions on credit and high heterogeneity was observed between countries (*I*^2^ >85% for all crop measures). However, in Burkina Faso, each additional decision on credit was positively associated with food crop count (mean difference 0·65 [0·24–1·05]), food group count (mean difference 0·33 [0·13–0·53]), and nutrient-dense crop cultivation (percentage point difference 11 [6–16]). Pooled associations using the binary indicator were similar (appendix p 24).

**Figure 4:**
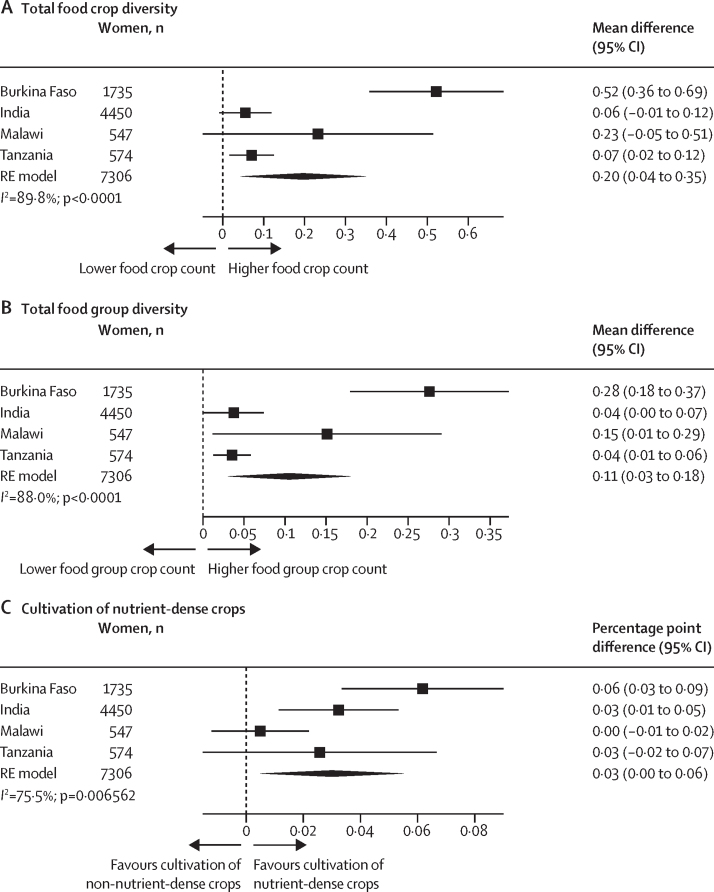
Association between group membership and total food crop diversity (A), total food group diversity (B), and cultivation of nutrient-dense crops (C) Total food group diversity ranged from zero to seven food groups. Cultivation of nutrient-dense crops was defined as the cultivation of at least one crop in the following food group categories: pulses, nuts and seeds, dark green leafy vegetables, other vitamin-A rich fruits and vegetables, other vegetables, or other fruit. Non-cultivators of nutrient-dense crops were those who grew only grains, roots, and tubers or if they grew no food groups (ie, not growing anything at all or only growing cash crops). Group membership was defined as the number of groups a woman was an active member of; if the woman reported no groups in their community, they were considered a member of zero groups. Country-specific adjusted estimates were pooled using an RE model using the DerSimonian and Laird method. The adjusted model for Burkina Faso controlled for intervention assignment, age, educational attainment, asset score, household size, and polygyny. The model for India controlled for intervention assignment, age, educational attainment, asset score, household size, and land size. The model for Malawi controlled for intervention assignment, age, educational attainment, asset score, and household size. The model for Tanzania controlled for intervention assignment, age, educational attainment, asset score, household size, and land size. RE=random-effects.

Group membership was associated with all three measures of crop diversity (figure 4). Heterogeneity was high (*I*^2^ >75%) for all crop diversity measures. Although active group membership was relatively low across most settings, with the exception of Malawi (appendix p 24), being an active member of one additional group was associated with 0·20 additional food crops (95% CI 0·04–0·35) and 0·11 additional food groups (0·03–0·18), but not with cultivation of nutrient-dense crops. For Malawi, an association was only found for food group count (mean difference 0·15 [0·01–0·29]). Results were consistent using the binary indicator of group membership (appendix p 25).

**Figure 5:**
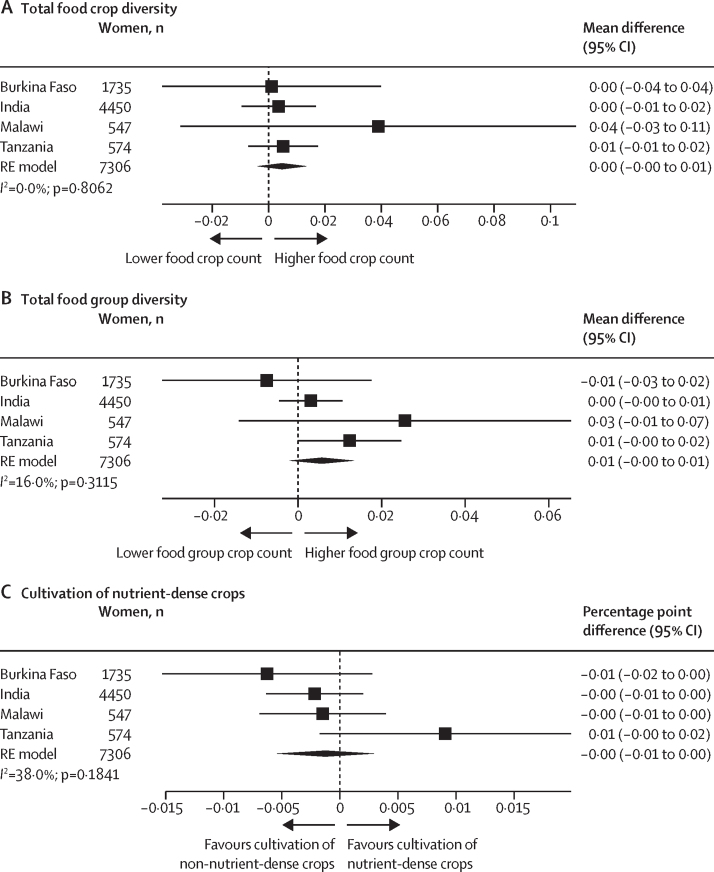
Association between productive work hours and total food crop diversity (A), total food group diversity (B), and cultivation of nutrient-dense crops (C) Total food group diversity ranged from zero to seven food groups. Cultivation of nutrient-dense crops was defined as the cultivation of at least one crop in the following food group categories: pulses, nuts and seeds, dark green leafy vegetables, other vitamin-A rich fruits and vegetables, other vegetables, or other fruit. Non-cultivators of nutrient-dense crops were those who grew only grains, roots, and tubers or if they grew no food groups (ie, not growing anything or only growing cash crops). Productive work hours were defined as the number of hours dedicated to productive work per day per individual; this version of the indicator does not include childcare. Country-specific adjusted estimates were pooled using an RE model using the DerSimonian and Laird method. The adjusted model for Burkina Faso controlled for intervention assignment, age, educational attainment, asset score, household size, and polygyny. The model for India controlled for intervention assignment, age, educational attainment, asset score, household size, and land size. The model for Malawi controlled for intervention assignment, age, educational attainment, asset score, and household size. The model for Tanzania controlled for intervention assignment, age, educational attainment, asset score, household size, and land size. RE=random-effects.

In the country-specific and pooled analyses, no association was identified between women's workload and crop diversity (figure 5). When using the binary indicator, work balance (defined as spending <10·5 h on productive work) was negatively associated with food group count in Malawi (mean difference –0·30 [95% CI –0·58 to–0·01]; appendix p 26). In sensitivity analyses, including childcare as productive work hours, work balance was negatively associated with food group count (–0·08 [–0·13 to –0·03]), driven by the association in Tanzania (appendix pp 26–27). The pooled association for work balance including childcare with food crop count was not significant; however, a negative association was observed in India (–0·19 [–0·35 to –0·02]; appendix p 28).

The direction of the associations did not substantially change when the association between women's empowerment and crop diversity was estimated using log-Poisson models (appendix pp 29–40).

## Discussion

We assessed country-specific and pooled associations between women's empowerment in agriculture and farm-level crop diversity in Burkina Faso, India, Malawi, and Tanzania. Among low-income agricultural households, women who partook in decision making across more areas, owned more assets, and actively participated in community groups were more likely to live in households cultivating a variety of crops, including nutrient-dense crops. These three domains are modifiable and, therefore, could be directly targeted by interventions. Associations varied by setting, indicating there might be nuanced country-specific interactions, underlying the importance of context in the measurement and interpretation of women's empowerment and benefits of diversifying cropping systems at the farm level.^38^

Our analysis builds on previous research by evaluating the association between domains of women's empowerment with types of farm-level crop diversity, and pooling associations across multiple countries. Consistent with previous studies from India and Bangladesh,^24^, ^25^ decision making, economic participation, and asset ownership were prominent determinants of crop diversity in our study. This finding is important considering the importance of crop diversity in potentially increasing and diversifying income, which might bolster livelihood resilience.^7^, ^9^, ^10^, ^14^ Crop diversity is also important because of its potential to improve access to diverse and nutritious foods, although the benefits of farm-level crop diversity for nutrition might be small in contexts with high market accessibility.^13^, ^14^ Crop diversity can be beneficial for planetary health; it can benefit natural systems, agrobiodiversity, soil health, and can serve as a sustainable pest and disease management tool that can prevent crop losses and potentially reduce pesticide use.^12^, ^39^

Many strategies exist for diversifying farm-level crop production, including agroforestry, intercropping, cover crops, crop rotations, and variety mixtures.^39^ In this analysis, data were only available on interspecific diversity (ie, different crops) and thus, we could not evaluate infraspecific diversity (ie, multiple varieties of the same crop). Future research should explore how women's empowerment is associated with the adoption of specific diversification strategies, which might have differential benefits on farmers’ nutritional and economic wellbeing and on planetary health. Moreover, we only evaluated on-farm diversification. Future studies should examine the optimal combination of crop and livelihood diversification for human and planetary health. Furthermore, future work should investigate the intensive margin, including crop productivity (eg, yield), since we only assessed the extensive margin (cultivation of nutrient-dense crops) in this study.

The results of our analysis provide other key insights. First, not all domains of women's empowerment in agriculture were associated with farm-level crop diversity. Although women's decision making, asset ownership, and participation in community groups were positively associated with crop diversity in most contexts, no consistent associations were identified with regard to credit or workload and crop diversity. Regarding credit, context was important: credit was associated with higher crop diversity in Burkina Faso and India, but not Malawi and negatively in Tanzania. Women with better access to credit and women who made more decisions on credit might divert their efforts towards other income-generating activities. Alternatively, credit might not empower women.^40^ Regarding workload, a larger work burden might translate to less capacity to diversify production. Or, higher crop diversity might increase work burden, consistent with previous explanations for negative and null associations between women's workload and nutritional outcomes,^25^ an explanation we cannot rule out due to the study design used. Second, women's empowerment domains were not associated with all crop diversity measures. For example, increased decision-making power was associated with the production of more food crops, but not with producing more nutritious crops. Although improving nutritious crop diversity is important for dietary quality and thus human health, improving food crop diversity is beneficial for planetary health, which encompasses both natural system and human health outcomes. Crop diversity was measured at the farm level and therefore we cannot confirm whether women were directly responsible for cultivation. However, in many contexts (including those studied in this analysis), plots are not divided on the basis of gender but rather are tended to by the same household, thus it is difficult to attribute a specific plot to cultivation by men or women.^41^ In the studied contexts, it is the agricultural-related activities themselves that are delegated by gender roles, which we were not able to distinguish using the available data.^42^

Our analysis had several limitations. First, gender norms and local interpretations of empowerment are subjective and differ across contexts,^43^ which could translate to differences in measurement even when using standardised tools, such as the WEAI. Second, because of our harmonisation, our final indicators deviate from the original WEAI, which might limit the comparability of our findings to other studies using the WEAI as designed. Third, we were unable to measure market access or crop sales income, which are important for diet quality and, in some cases, agricultural diversification.^14^, ^44^ Fourth, we were unable to evaluate gender parity due to a paucity of data, but hypothesise that the associations might have been even larger with an increase in gender parity due to greater collective decision making and cooperation. Fifth, our analyses were cross-sectional, which limited our ability to identify causal effects and rule out reverse causality. Sixth, our sample only included women of reproductive age with young children, which might limit the generalisability of our findings. Further research should examine these relationships longitudinally and among other populations of women, such as those without children or with older children. Women with older children might be less burdened by childcare, and therefore, more empowered in terms of work balance. However, we did not find an association between work balance and crop diversity. Additionally, non-subsistence farmers or those with greater market engagement might have more access to more agricultural services, which could moderate the associations between women's empowerment and crop diversity.

In practice, nutrition-sensitive agricultural interventions can improve nutrition outcomes by improving women's empowerment.^45^ Other interventions successful at improving women's empowerment, particularly the modifiable domains we identified, include economic strengthening interventions^46^ and promoting active group membership.^47^ However, evidence on women's groups indicates that context is important, even within countries.^28^, ^47^ More work is needed to understand what type of community groups can improve both women's empowerment and crop diversity.

Other structural aspects of women's empowerment, such as gender and patriarchal norms, should also be considered.^46^, ^47^ More research is needed on how to simultaneously improve women's empowerment and promote crop diversity. In future research it will be important to identify how women's empowerment can be fostered in a way that does not add to women's existing work burdens and responsibilities, and how to support households to diversify production considering the risks and investments involved in growing new crops.

In conclusion, we found that women's input into productive decisions, asset ownership, and group membership were associated with increased farm-level crop diversity. Our findings highlight the importance of, and heterogeneity in, women's empowerment as a potential pathway to increase farm-level crop diversity, improve food systems resilience, and achieve the balance between a healthy diet and a healthy planet.

## Data sharing

Data for the Burkina Faso and Malawi studies are available on reasonable request to Aulo Gelli (a.gelli@cgiar.org), the principal investigator of the studies. Data for the India study will be available on the London School of Hygiene & Tropical Medicine Data Compass. Data on the India study is available upon request to Suneetha Kadiyala (suneetha.kadiyala@lshtm.ac.uk), the principal investigator of the study. Data on the Tanzania study are available on request to Sera Young (sera.young@northwestern.edu), the principal investigator of the study.

## Declaration of interests

We declare no competing interests.
