# Supplementary material for: Women's empowerment, production choices, and crop diversity in Burkina Faso, India, Malawi, and Tanzania: a secondary analysis of cross-sectional data
Source: Lancet Planet Health. 2023 Jul 10;7(7):e558–69. doi: 10.1016/S2542-5196(23)00125-0 (PMC10352962; doi:10.1016/S2542-5196(23)00125-0)
Supplement: Supplementary appendix [file mmc1.pdf]

THE LANCET
Planetary Health

Supplementary appendix

This appendix formed part of the original submission and has been peer reviewed. We post it as supplied by the authors.

Supplement to: Connors K, Jaacks LM, Awasthi A, et al. Women's empowerment, production choices, and crop diversity in Burkina Faso, India, Malawi, and Tanzania: a secondary analysis of cross-sectional data. *Lancet Planet Health* 2023; **7:** e558–69.

Supplementary Material for

Women's Empowerment, Production Choices, and Crop Diversity: a secondary analysis of cross-sectional data from four low- and middle-income countries

Kaela Connors, MSc[1,2], Prof Lindsay M. Jaacks, PhD[1,2], Ananya Awasthi, MPH[3], Karoline Becker, MPhil[4], Prof Rachel Bezner Kerr, PhD[5], Emily Fivian, MPH[6], Aulo Gelli, PhD[7], Helen Harris-Fry, PhD[6], Jessica Heckert, PhD[7], Prof Suneetha Kadiyala, PhD[6], Elena Martinez, MS[8], Marianne V. Santoso, PhD[9], Sera L. Young, PhD[10], Lilia Bliznashka, PhD[2,7]*

[1]Harvard T.H. Chan School of Public Health, Boston, MA, USA
[2] Global Academy of Agriculture and Food Systems, The University of Edinburgh, Midlothian, UK
[3]Anuvaad Solutions, New Delhi, India
[4] Department of International Development, University of Oxford, Oxford, UK
[5] Department of Global Development, Cornell University, NY, USA
[6] Department of Population Health, London School of Hygiene & Tropical Medicine, London, UK
[7] International Food Policy Research Institute, Washington, DC, USA
[8] Friedman School of Nutrition Science and Policy at Tufts University, Boston, MA, USA
[9]Department of Global Development, Cornell University, NY, USA
[10]Department of Anthropology, Northwestern University, Evanston, IL, USA

***Correspondence:**
l.bliznashka@cgiar.org
1201 I St NW, Washington, DC 20001, USA
+1-202- 862-6493

Contents

Supplementary Methods 5
Theory of Change 5
Figure S1 – Pathways from women's empowerment in agriculture to crop diversity 5
Women's empowerment measurement 6
Modifications to A-WEAI indicators for harmonisation across countries 7
Supplementary References 8
Table S1 – Comparison of characteristics across studies 9
Table S2 – Items included in A-WEAI indicators across studies 11
Table S2 (continued) – Items included in A-WEAI indicators across studies 12
Table S3 – Categorisation of crops into food groups according to FAO guidelines for calculating Women's Minimum Dietary Diversity (MDD-W) per study 13
Table S3 (continued) – Categorisation of crops according to Women's Minimum Dietary Diversity (MDD-W) per study 14

Table S4 - Assets included in Asset Score for each study 15
Table S5 – Summary of incomplete information on women's empowerment in agriculture and cropping patterns for each study 16
Table S6 – Summary of sociodemographic characteristics across samples 17
Table S7 – Summary of women's empowerment count indicators across samples 18
Table S8 – Summary of women's empowerment binary indicators across samples 19
Table S9 - Summary of workload indicators including childcare across samples 20
Figure S2 – Cultivation of food groups by country 21
Figure S3 – Crop diversity by country 21
Figure S4 – Input into productive decisions and crop diversity 22
Figure S5 – Ownership of assets and crop diversity 23
Figure S6 – Access to and decisions on credit and crop diversity 24
Figure S7 – Group membership and crop diversity 25
Figure S8 – Work balance and crop diversity 26
Figure S9 – Productive work hours including childcare and crop diversity 27
Figure S10 – Work balance including childcare and crop diversity 28
Figure S11 –Input into productive decisions and crop diversity 29
Figure S12 – Ownership of Assets and Crop Diversity 30
Figure S13 – Access to and Decisions on Credit and Crop Diversity 31
Figure S14 – Group Membership and Crop Diversity 32
Figure S15 – Productive Work Hours and Crop Diversity 33
Figure S16 – Input into productive decisions and crop diversity 34
Figure S17 – Ownership of assets and crop diversity 35
Figure S18 – Access to and decisions on credit and crop diversity 36
Figure S19 – Group membership and crop diversity 37
Figure S20 – Work balance and crop diversity 38
Figure S21 – Productive work hours including childcare and crop diversity 39
Figure S22 – Work balance including childcare and crop diversity 40
Supplementary workbook 1 Women's empowerment and crop diversity in Burkina Faso 41
- **Table S10 Input into productive decisions and crop diversity in Burkina Faso** 41
- **Table S11 Input into productive decisions and crop diversity in Burkina Faso** 41
- **Table S12 Ownership of Assets and Crop Diversity in Burkina Faso** 41
- **Table S13 Ownership of Assets and Crop Diversity in Burkina Faso** 41
- **Table S14 Ownership of Agricultural Assets and Crop Diversity in Burkina Faso** 41
- **Table S15 Access to and decisions about credit and Crop Diversity in Burkina Faso** 41

Table S16 Access to credit and Crop Diversity in Burkina Faso 41
Table S17 Decisions on credit and Crop Diversity in Burkina Faso 41
Table S18 Group Membership and Crop Diversity in Burkina Faso 41
Table S19 Group Membership and Crop Diversity in Burkina Faso 41
Table S20 Work Balance and Crop Diversity in Burkina Faso 41
Table S21 Productive Work Hours and Crop Diversity in Burkina Faso 41
Table S22 Work Balance including childcare and Crop Diversity in Burkina Faso 41
Table S23 Productive Work Hours including childcare and Crop Diversity in Burkina Faso 41
Supplementary workbook 2 Women's empowerment and crop diversity in India 41
Table S24 Input into productive decisions and crop diversity in India 41
Table S25 Input into productive decisions and crop diversity in India 41
Table S26 Ownership of Assets and Crop Diversity in India 41
Table S27 Ownership of Assets and Crop Diversity in India 41
Table S28 Ownership of Agricultural Assets and Crop Diversity in India 41
Table S29 Access to and decisions about credit and Crop Diversity in India 41
Table S30 Access to credit and Crop Diversity in India 41
Table S31 Decisions on credit and Crop Diversity in India 41
Table S32 Group Membership and Crop Diversity in India 41
Table S33 Group Membership and Crop Diversity in India 41
Table S34 Work Balance and Crop Diversity in India 41
Table S35 Productive Work Hours and Crop Diversity in India 41
Table S36 Work Balance including childcare and Crop Diversity in India 41
Table S37 Productive Work Hours including childcare and Crop Diversity in India 41
Supplementary workbook 3 Women's empowerment and crop diversity in Malawi 41
Table S38 Input into productive decisions and crop diversity in Malawi 41
Table S39 Input into productive decisions and crop diversity in Malawi 41
Table S40 Access to and decisions about credit and Crop Diversity in Malawi 41
Table S41 Access to credit and Crop Diversity in Malawi 41
Table S42 Decisions on credit and Crop Diversity in Malawi 41
Table S43 Group Membership and Crop Diversity in Malawi 41
Table S44 Group Membership and Crop Diversity in Malawi 41
Table S45 Work Balance and Crop Diversity in Malawi 41
Table S46 Productive Work Hours and Crop Diversity in Malawi 41
Supplementary workbook 4 Women's empowerment and crop diversity in Tanzania 42

Table S47 Input into Productive Decisions and Crop Diversity in Tanzania 42
Table S48 Input into Productive Decisions and Crop Diversity in Tanzania 42
Table S49 Access to and decisions on credit and Crop Diversity in Tanzania................. 42
Table S50 Access to and decisions on credit and Crop Diversity in Tanzania................. 42
Table S51 Group Membership and Crop Diversity in Tanzania 42
Table S52 Group Membership and Crop Diversity in Tanzania 42
Table S53 Work balance and crop diversity in Tanzania 42
Table S54 Productive work hours and Crop Diversity in Tanzania 42
Table S55 Work balance including childcare and crop diversity in Tanzania 42
Table S56 Productive work hours including childcare and crop diversity in Tanzania .. 42

Supplementary Methods

Theory of Change

Figure S1 – Pathways from women's empowerment in agriculture to crop diversity

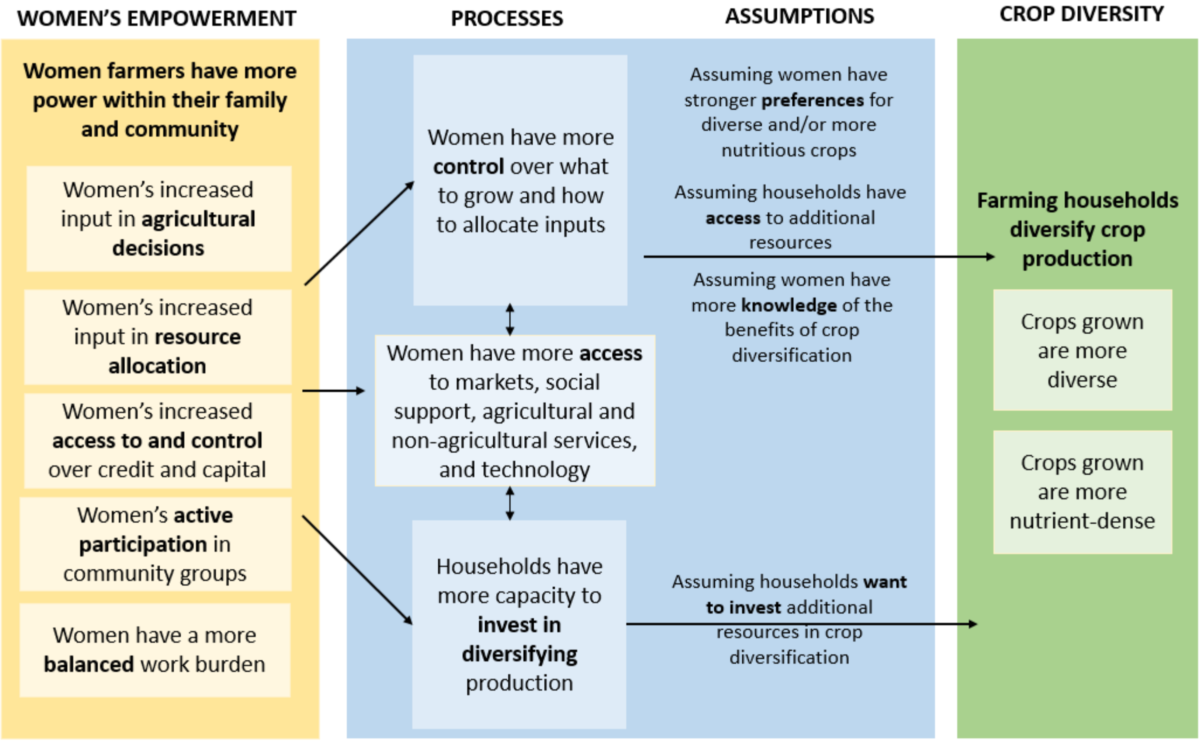

We theorise that the relationship between women's empowerment in agriculture and crop diversity can be explained through the processes depicted in **Figure S1:** (1) increased women's control over what to grow and how to allocate inputs, (2) increased access to markets, social support, agricultural services (including crop insurance), non-agricultural services, and technology, and (3) increased household capacity to invest in diversified production. Women's empowerment in agriculture is comprised of four key domains: increased input in agricultural decisions, increased input in resource allocation, increased access and control over credit and capital, increased participation in community groups, and more balanced work load.[1] Improving women's asset ownership which is a part of access to productive capital, access to knowledge, and access to resources can increase their household bargaining power, and thereby, grant them greater agency and ability to negotiate roles in household decision-making.[2] Women's participation in community groups can lead to women's increased access to agricultural and nutritional information [3], as well as the redistribution of labour allowing them more time for farming (although this may be context-dependent given than empowerment may increase participation in off-farm work).[2,4]

Empowerment in these domains may increase crop diversity through a number of mechanisms. When women acquire the agency and resources to do so, they may allocate land, labour, and other inputs to focus on producing diverse and nutrient-rich crops.[4] This is reflected in evidence showing (which is context-dependent) that women are typically more responsible for nourishing their families and typically focus on food cropping, whilst men are often more responsible for providing income for the family and invest more in cash cropping.[5] In rural Africa, women have been found to possess unique agricultural knowledge apart from men such as with respect to seed selection, planting, harvesting, cleaning, and storing.[6] This knowledge is shared among women within communities and intergenerationally which contributes to the preservation of a myriad endemic and wild crops.[6] Women also tend to make decisions that support healthier and more nutritious food choices for themselves and their families, reflective of their roles as caregivers and food provisioners.[1, 8–11] A study in Malawi found that the association between farm-level crop diversity and household

dietary diversity was stronger in female-headed households compared to male-headed households.[12] This supports the idea that women may be more motivated to diversify agricultural production in order to benefit household nutrition.

Empowerment of women may also lead to crop diversity through benefits to the household such as increased capacity to diversify production, given women's empowerment has been linked to improving household economic outcomes.[2,4] A study evaluating crop diversification among smallholder farms in Vietnam found that higher levels of women's education, a proxy for women's empowerment in this study, had a greater impact on agricultural technical efficiency than men's education.[13] It also found that technical efficiency improved with greater crop diversity as opposed to mono-cropping farms.[13]

Moreover, empowerment can increase women's and household's use of agricultural technology, market participation, and utilisation of agricultural programmes such as crop insurance which can mediate the association between women's empowerment and crop diversity.[4,14] A study assessing the adoption of agricultural technology that involved intercropping in Western Kenya found that female-headed households were more likely to adopt the technology than male-headed households.[15] Further, women's empowerment may increase women's engagement with markets. A study conducted in Maharashtra, India found that greater household market orientation was associated with greater women's empowerment in agriculture.[16] Although much of the literature focusses on how markets can serve to empower women, the reverse is also plausible where women that are more empowered with greater access to resources and greater mobility may have increased engagements with markets, which in turn can influence crop diversity such that women change their crop portfolio to respond to market demand. Lastly, services such as crop insurance may provide smallholder farmers financial security against crop failures; women's empowerment may reduce barriers to their uptake of these services and enable them to adopt new agricultural practices and cultivate new (potentially riskier) crops.[17] In all, these pathways illustrate that, if women have the power to, may influence how their households allocate their land, labour, and other inputs differently to men, with more focus on producing more, and more nutrient-rich crops.

Women's empowerment measurement

Women's empowerment has been previously defined by Kabeer as the 'ability to exercise choice' which is conceptualised within the realms of resources, agency, and achievements.[18] Resources extend beyond the material and may constitute immaterial resources such as social support. Per Kabeer's definition, agency refers to the ability to make decisions and participate in negotiation processes. Measuring 'achievements' in both these realms depends on the reference and context in which they are analysed. Differences in choice preference do not always translate to gender inequality, and therefore, empirical measurements of empowerment need to reflect this. [18] In another prominent framework, Narayan defines empowerment based on principles of self-determination and the 'freedom of choice'.[19] Empowerment relies on four key universal elements: access to information, inclusion of marginalised groups, social, political, and public accountability, and lastly, local community organisation and mobilisation.[19] Lastly, in another important body of work, Alsop *et al.* define empowerment as 'the process of…transforming choices into desired actions and outcomes'.[20] This definition emphasises the synergies between 'agency' and 'structure'; fostering agency and structural opportunities support the ability to make and act on choices that translate empowerment-related outcomes.[20]

The concepts of agency and exercising of choice are shared across all three of these frameworks. The Women's Empowerment in Agriculture Index (WEAI) operationalises the measurement of empowerment by drawing from elements of these definitions.[1] The WEAI uses Kabeer's conceptualisation of agency which includes decision making and negotiation processes measured as input into productive decisions on agriculture and access to and control over assets, income, and credit.[18] Alsop *et al.* underpin measurement of these areas of achievement as well, adding capability which means the capacity to act on and carry out one's decisions to fruition.[20] Narayan's definition importantly extends the measurement of empowerment to the group or community level.[19] Therefore, the WEAI measures women's participation in community groups and leadership as another central aspect of empowerment. Lastly, time poverty inhibits individuals from actualising their choices and freedom as defined by these three definitions of empowerment. As such, the WEAI measures time allocation as another means by which they may achieve empowerment and ability to engage in other activities of their choice.

We used two specific versions of the WEAI the build off the original version: the abbreviated WEAI (A-WEAI), which shortened the original WEAI by dropping some indicators, and the project-level WEAI (pro-WEAI), which

builds off the A-WEAI and adds additional indicators.[21] Importantly, the A-WEAI questionnaire is fully embedded in the pro-WEAI questionnaire. The A-WEAI was used in India, Malawi, and Tanzania and the pro-WEAI in Burkina Faso. Data on the control over use of income domain were not collected in India and data on asset ownership were not available in Tanzania and Malawi. We took information from each study that could be used to calculate the A-WEAI indicators to enable comparisons and pooling across studies. Another motivation for analysing individual empowerment indicators was to expand knowledge beyond a composite index and identify the specific aspects of women's empowerment that contribute to crop diversity.[9]

Modifications to A-WEAI indicators for harmonisation across countries

The following modifications were made to harmonise the binary indicators across studies. First, empowerment in input into productive decisions was defined as having 'at least one area in which the respondent had some input in decisions, or felt they could make decisions, or was the sole decision maker'.[22] However, there was insufficient variability in India [4,343 (97.6%) met condition] and Tanzania [563 (98.0%) met condition]. Therefore, the threshold was adjusted to 'at least two areas'. Second, the indicator for access to and decisions on credit is calculated based on having at least one credit/loan source that anyone in the respondent's household took out 'and' that the respondent participated in decisions on.[22] However, only 5% met this condition in India and no one met this condition in Tanzania. Therefore, we replaced the 'and' with an 'or' to make the statement more inclusive. Third, the study in India did not collect information on whether the household had access to credit/loan sources. Therefore, the follow-up question on whether anyone in the respondent's household borrowed from that credit source in the last 12 months was used as a proxy for access to calculate the binary indicator. Fourth, participating in decisions on credit was originally defined as being a 'sole' or 'joint' decision-maker.[22] However, the study in Tanzania did not collect information on joint decision-making about whether to borrow or how to use the credit. As a result, decisions on credit were restricted to 'sole' decision-maker for that credit source in all studies. Lastly, the definitions for empowerment in time allocation differ between the A-WEAI and pro-WEAI. Specifically, the pro-WEAI includes childcare as productive work while the A-WEAI does not.[21,22] We used the A-WEAI definitions for our primary analyses and assessed the association with the pro-WEAI version of workload in sensitivity analyses given that only 111 (2.5%) of women in India met the criteria for work balance using this definition. For this, we added the time spent on childcare multiplied by 0.5 to the sum of productive work hours.

Supplementary References

1. Alkire, S., Meinzen-Dick, Ruth, Peterman, Amber, Quisumbing, Agnes R. & Seymour, G. *The women's empowerment in agriculture index*. (Oxford Poverty & Human Development Initiative, 2013).
2. Doss, C. Intrahousehold Bargaining and Resource Allocation in Developing Countries. *The World Bank Research Observer* **28**, 52–78 (2013).
3. Meinzen-Dick, R., Behrman, J. A., Pandolfelli, L., Peterman, A. & Quisumbing, A. R. Gender and Social Capital for Agricultural Development. in *Gender in Agriculture: Closing the Knowledge Gap* (eds. Quisumbing, A. R. et al.) 235–266 (Springer Netherlands, 2014). doi:10.1007/978-94-017-8616-4_10.
4. Anderson, C. L., Reynolds, T. W., Biscaye, P., Patwardhan, V. & Schmidt, C. Economic Benefits of Empowering Women in Agriculture: Assumptions and Evidence. *The Journal of Development Studies* **57**, 193–208 (2021).
5. Carr, E. R. Men's Crops and Women's Crops: The Importance of Gender to the Understanding of Agricultural and Development Outcomes in Ghana's Central Region. *World Development* **36**, 900–915 (2008).
6. Hosken, L. The critical role that African rural women play as custodians of seed diversity and wild relatives in the context of climate change. *Biodiversity* **18**, 98–101 (2017).
7. Nordhagen, S., Pascual, U. & Drucker, A. G. Gendered differences in crop diversity choices: A case study from Papua New Guinea. *World Development* **137**, 105134 (2021).
8. Santoso, M. V. *et al.* Role of Women's Empowerment in Child Nutrition Outcomes: A Systematic Review. *Advances in Nutrition* **10**, 1138–1151 (2019).
9. Quisumbing, A. R., Sproule, K., Martinez, E. M. & Malapit, H. Do tradeoffs among dimensions of women's empowerment and nutrition outcomes exist? Evidence from six countries in Africa and Asia. *Food Policy* **100**, 102001 (2021).
10. Sraboni, E., Malapit, H. J., Quisumbing, A. R. & Ahmed, A. U. Women's Empowerment in Agriculture: What Role for Food Security in Bangladesh? *World Development* **61**, 11–52 (2014).
11. Malapit, H. J. L. & Quisumbing, A. R. What dimensions of women's empowerment in agriculture matter for nutrition in Ghana? *Food Policy* **52**, 54–63 (2015).
12. Jones, A. D., Shrinivas, A. & Bezner-Kerr, R. Farm production diversity is associated with greater household dietary diversity in Malawi: Findings from nationally representative data. *Food Policy* **46**, 1–12 (2014).
13. Nguyen, H. Q. Analyzing the economies of crop diversification in rural Vietnam using an input distance function. *Agricultural Systems* **153**, 148–156 (2017).
14. Fletschner, D. & Kenney, L. Rural Women's Access to Financial Services: Credit, Savings, and Insurance. in *Gender in Agriculture: Closing the Knowledge Gap* (eds. Quisumbing, A. R. et al.) 187–208 (Springer Netherlands, 2014). doi:10.1007/978-94-017-8616-4_8.
15. Khan, Z. R., Amudavi, D. M., Midega, C. A. O., Wanyama, J. M. & Pickett, J. A. Farmers' perceptions of a 'push–pull' technology for control of cereal stemborers and Striga weed in western Kenya. *Crop Protection* **27**, 976–987 (2008).
16. Gupta, S., Pingali, P. L. & Pinstrup-Andersen, P. Women's empowerment in Indian agriculture: does market orientation of farming systems matter? *Food Sec.* **9**, 1447–1463 (2017).
17. Born, L., Spillane, C. & Murray, U. Integrating gender into index-based agricultural insurance: a focus on South Africa. *Development in Practice* **29**, 409–423 (2019).
18. Kabeer, N. Resources, Agency, Achievements: Reflections on the Measurement of Women's Empowerment. *Development and Change* **30**, 435–464 (1999).
19. Narayan, D., Stern, N., Nankani, G., Page, J. & Jorgensen, S. *Empowerment and poverty reduction: a source book*. (PREM, 2002).
20. Alsop, R., Bertelsen, M. F. & Holland, J. *Empowerment in Practice: From Analysis to Implementation*. (World Bank Publications, 2006).
21. Malapit, H. *et al.* Development of the project-level Women's Empowerment in Agriculture Index (pro-WEAI). *World Development* **122**, 675–692 (2019).
22. Malapit, H. J. *et al. The Abbreviated Women's Empowerment in Agriculture Index (A-WEAI)*. https://papers.ssrn.com/abstract=3012806 (2017).
23. Bandyopadhyay, A., Haile, B., Azzarri, C. & Somé, J. Analyzing the Drivers of Household Dietary Diversity: Evidence from Burkina Faso. *Food Nutr Bull* **42**, 530–550 (2021).
24. Human Rights Watch. Burkina Faso: Events of 2022. in *World Report 2023* (2023).
25. Koppmair, S., Kassie, M. & Qaim, M. Farm production, market access and dietary diversity in Malawi. *Public Health Nutrition* **20**, 325–335 (2017).
26. DHS. Guide to DHS-7 Statistics. https://www.dhsprogram.com/publications/publication-dhsg1-dhs-questionnaires-and-manuals.cfm (2020).

Table S1 – Comparison of characteristics across studies

	Setting	Agroecosystem and Region	Agricultural practices	Sociocultural context	Spousal characteristics	Market Engagement*
**Burkina Faso**	Rural and peri-urban communities	Boucle de Mouhoun<br>Centre-Ouest<br>Haut-Bassins<br><br>Hot, dry and monsoon season<br><br>Some parts are tropical	Husbands are typically responsible for the cultivation of staple crops and are supported by their wives' labor. Wives are often allocated a small share of land or space for a kitchen garden and are responsible for cultivating condiments (e.g., greens, vegetables, spices).	Patrilineal society with high polygyny	Not collected	Smallholder farmers often rely on markets to purchase at least some of the food households consume.[23]<br><br>Market access is often limited with approximately 50% of households traveling for longer than 60 minutes to reach markets.[23]<br><br>Frequent political unrest further interferes with market access by limiting people's ability to travel between villages.[24]<br><br>In this sample, we were not able to calculate the proportion of crops sold out of those produced. However, we did calculate the proportion of households selling any amount of crop (77.6% of households sold some type of crop).<br><br>The proportion sold out of what was produced was:<br>Grains, roots, and tubers 39.7%<br>Pulses 17.9%<br>Nuts and seeds 48.7%<br>Vegetables 11.4%<br>Dark green leafy vegetables 1.5%<br>Vitamin A rich vegetables 0.4%<br>Fruits 0.2%<br>Cash crops 32.6%
**India**	Rural agricultural villages	Keonjhar, Odisha<br><br>Forests, temperate with warm summers	Men and women typically cultivate the same plot but are responsible for different tasks. For example, men are often responsible for mixing and spraying chemicals, whereas women are responsible for harvesting.	Majority of households contain both male and female<br><br>Of the final analytic sample (n = 1,735), 2,606 (58·6%) belong to the Scheduled Tribe, 402 (9·0%) belonged to the Scheduled Case, 1,335 (30·0%) belonged to the Other Backward Caste, and 107 belong to none of the above (2·4%).	mean(sd) based on n = 3,606<br><br>Age: 30.3 (6.2) years<br>Years of formal education completed: 7.3 (4.2)<br><br>Regression results do not change when adjusted for spousal characteristics	Market participation very low (≤ 10% of quantity produced of crops by type was sold).<br><br>The proportion sold out of what was produced was:<br>Grains, roots, and tubers 9.9%<br>Pulses 3.7%<br>Vegetables 14.5%<br>Green leafy vegetables 13.0%<br>Vitamin A rich vegetables 17.5%<br>Fruits 13.3%<br>Cash crops 5.2%
**Malawi**	Rural, agricultural communities, with a history of food insecurity	Zomba district<br><br>Woodlands and grasslands	Community Based Childcare Centre (CBCC) gardens, demonstration fields, home-gardens	Matrilineal kinship systems (land inherited following the female lineage, uxorilocal settlement, but not necessarily passed on to women; husband	Not collected	Limited market access with approximately 40% of villages not having a market in the community and average travel time to the district market of approximately 1.4 hours.[25]<br><br>Limited market engagement, with up to 30% of food crops sold at markets.[25]

				moves to wife's village after marriage)		
**Tanzania**	Rural agricultural villages – among the poorest and most food insecure in Tanzania	Singida district, Singida region<br><br>Semi-arid, suffers recurrent drought	Smallholder farms, mixed crop-livestock systems	Patrilineal system with high levels of gender inequity. High levels of male migration to cities for work.<br><br>Of the final analytic sample (n = 574), 552 (96·3%) belong to the Nyaturu tribe, 13 (2·3%) belong to the Nyiramba tribe, and 9 (1·4%) belong to 'other' tribe.	Not collected	Dependence on subsistence farming, relatively easy access to markets for non-lucrative cash crops (i.e. onions, sunflowers) and low income.

*For Burkina Faso, we only had data on whether households sold any crops or not. For India, we had data on the quantity of crops sold and the quantity of crops produced. For both, we do not have quantities or where it was sold (market, vendor, and neighbour) which are all important to understand market engagement. Information on crop utilisation or market engagement was not available for Malawi nor Tanzania.

Table S2 – Items included in A-WEAI indicators across studies

	Burkina Faso	India	Malawi	Tanzania
**Input in Productive Decisions***	• Staple grain farming<br>• Horticultural (gardens)<br>• Fishpond<br>• Non-farm economic activities<br>• Wage and salary employment<br>• Large, occasional household purchases<br>• Routine household purchases	• Food production for household consumption: crops, livestock, or fish etc. that are grown for household food consumption<br>• Cash crop farming<br>• Livestock raising<br>• Non-farm economic activities<br>• Minor Household Expenditures	• Food crop farming for household (crops)<br>• Cash crop farming<br>• Livestock raising<br>• Non-farm economic activities<br>• Wage and salary employment<br>• Fish or fishpond culture<br>• Major household expenditures<br>• Minor household expenditures	• Food Crop Farming*<br>• Cash Crop farming<br>• Livestock Raising<br>• Non-farm economic activities<br>• Wage and salary employment<br>• Major household expenditures<br>• Minor household expenditures
**Ownership of Assets†**	• Agricultural land<br>• Large livestock<br>• Small livestock<br>• Fishpond<br>• Non-mechanised farm equipment<br>• Mechanised farm equipment<br>• House or building<br>• Large consumer durable<br>• Small consumer durable<br>• Cell phone<br>• Other non-ag land<br>• Transportation	• Agricultural land<br>• Large livestock<br>• Small Livestock<br>• Non-mechanised farm equipment<br>• Mechanised farm equipment<br>• House or building<br>• Non-farm business equipment<br>• Large consumer durable<br>• Small consumer durable<br>• Cell phone<br>• Other non-ag land<br>• Jewellery	• Large livestock<br>• Small livestock<br>• Bees/beehives<br>• Farm equipment (mechanised)<br>• Nonfarm business equipment<br>• House or other structures<br>• Small consumer durables<br>• Cell Phone<br>• Other land not used for agriculture<br>• Means of transportation	*Not collected*

Table S2 (continued) – Items included in A-WEAI indicators across studies

	Burkina Faso	India	Malawi	Tanzania
**Access to and decisions on credit**	• NGO<br>• Formal lender<br>• Informal lender<br>• Friends/relatives<br>• Group based micro-finance<br>• Informal credit/savings	• Formal Lender<br>• Informal Lender<br>• Friends/relatives	• NGO<br>• Formal lender<br>• Informal Lender<br>• Friends/relatives<br>• Group-based micro-finance<br>• Informal credit/savings groups	• NGO<br>• Formal Lender<br>• Informal Lender<br>• Friends/relatives<br>• Group-based lending<br>• Informal credit/savings groups
**Group Membership**	• Agricultural / livestock / fisheries producer's<br>• Water users' group<br>• Forest users' group<br>• Credit or microfinance group<br>• Mutual help or insurance group<br>• Trade and business association group<br>• Civic group (improving community) or charitable group (helping others)<br>• Religious group	• Farmer's (NABARD) club<br>• Water group<br>• Forest Group<br>• Credit/micro-finance group<br>• Village Development group<br>• Religious Group<br>• SHG<br>• School-based group<br>• Nutrition-related group<br>• Youth Club	• Agricultural/livestock/fisheries producer's groups<br>• Water user's group<br>• Forest or other natural resource user's group<br>• Credit or microfinance group<br>• Civic/community groups<br>• Religious group<br>• Other farmer-based organisations<br>• Other women's/men's group	*Not collected*
**Workload**	• Sleeping or resting<br>• Eating or drinking<br>• Personal care<br>• School (including homework)<br>• Wage labour<br>• Wage labour for own business<br>• Agricultural labour (cultivating basic cereals)<br>• Working in the garden of cultivating products of high agricultural value<br>• Large Livestock raising (oxen or donkeys)<br>• Small livestock raising (sheep, pigs, or goats)<br>• Poultry raising (chickens or guinea fowl)<br>• Fishpond culture<br>• Shopping or obtaining services such as healthcare<br>• Weaving, sewing, or making textiles<br>• Food preparation<br>• Domestic work (e.g., collecting water, wood, or other loads)<br>• Childcare<br>• Travel (not for work or school)<br>• Sport activities<br>• Social and leisure activities<br>• Religious activities	• Sleeping or resting<br>• Eating, drinking or other relaxing leisure activities including social or religious activities<br>• Personal care (e.g., dressing, showering)<br>• School/studies<br>• Light non-farm wage labour<br>• Physically strenuous non-farm wage labour<br>• Traveling, communing<br>• Heavy, strenuous agricultural work<br>• Mild/moderately strenuous agricultural work<br>• Livestock raising or fishpond culture<br>• Foraging or hunting<br>• Collecting water, wood, or other loads<br>• Food preparation<br>• Childcare<br>• Defecation	• Labour<br>• Farm<br>• Construction<br>• Shopping<br>• Housework<br>• Travel<br>• Care<br>• Leisure<br>• Religious<br>• Personal<br>• School<br>• Sleep	• Heavy housework<br>• Light housework<br>• Childcare<br>• Leisure<br>• Sleep<br>• Unknown

*For Tanzania, the following items were combined with food crop farming: 1) sorghum, maize, and millet farming, 2) legumes farming, 3) other food crop farming for household consumption.
†For Burkina Faso, the following items were combined with small livestock: 1) pigs, and 2) poultry. For Malawi, the following items were combined with small livestock: 1) small livestock, and 2) fowl.

Table S3 – Categorisation of crops into food groups according to FAO guidelines for calculating Women's Minimum Dietary Diversity (MDD-W) per study

	Burkina Faso	India	Malawi	Tanzania
Grains, roots, and tubers	• Corn<br>• Sorghum<br>• Millet<br>• Rice<br>• Yam<br>• White Sweet potato<br>• Cassava	• Maize<br>• Other white tuber<br>• Millet<br>• Other cereal<br>• Other non-white Tuber<br>• Paddy (rice)<br>• Potato<br>• Elephant foot yam<br>• Yam<br>• Ragi (finger millet)	• Maize<br>• Finger Millet<br>• Sorghum<br>• Cassava<br>• White Sweet Potato<br>• Irish potato<br>• Plaintain<br>• Soyabean flour<br>•	• Sorghum<br>• Maize<br>• Finger millet<br>• Pearl millet<br>• Cassava<br>• Wheat<br>• Amaranth<br>• Potato<br>• Rice<br>• Girigirani<br>• Mihogo<br>• Cassava
Pulses (beans, peas, and lentils)	• Cowpea/bean<br>• Soybean	• Butter beans<br>• Red gram<br>• Black gram<br>• Cluster beans<br>• Green gram<br>• Horse gram<br>• Lentil<br>• Yardlong beans<br>• Other gram	• Bean (brown)<br>• Pigeonpea<br>• Cowpea	• Cowpea<br>• Pigeon pea<br>• Lablab<br>• Soy<br>• Lentils<br>• Choroko<br>• Beans
Nuts and seeds	• Sesame<br>• Peanut<br>• Ground pea	• Cashew<br>• Groundnut<br>• Other seed, nut, or spice<br>• Sesame<br>• Sunflower seed<br>• Linseed	• Groundnut	• Sunflower<br>• Groundnut<br>• Bambara nut
Dark green leafy vegetables	• Spinach<br>• Salad<br>• Other cultivated leaves	• Other GLV<br>• Spinach<br>• Green amaranth<br>• Indian spinach<br>• Spinage<br>• Drumstick	• Tanaposi<br>• Nkhwani<br>• Other cultivated green leafy vegetables	• Spinach
Other vitamin A rich fruits and vegetables	• Mango<br>• Carrot<br>• Squash	• Pumpkin<br>• Sweet potato	• Orange Sweet Potato<br>• Pumpkin	• Pumpkin<br>• Sweet potato
Other vegetables	• Sauerkraut<br>• Zucchini<br>• Tomato<br>• Eggplant<br>• Onion<br>• Pepper<br>• Cucumber<br>• Green beans<br>• Okra<br>•	• Cauliflower<br>• Chillies<br>• Green beans<br>• Green peas<br>• Zucchini<br>• Cucumber<br>• Mushroom<br>• Jhudang<br>• Onion<br>• Other vegetable<br>• Radish<br>• Capsicum<br>• Okra<br>• Cabbage<br>• Tomato<br>• Brinjal	• Onion<br>• Cabbage<br>• Tomato<br>• Cucumber<br>• Okra<br>• Other vegetables<br>•	• Okra<br>• Cabbage<br>• Chinese cabbage<br>• Onion<br>• Eggplant<br>• Tomato<br>•

Table S3 (continued) – Categorisation of crops according to Women's Minimum Dietary Diversity (MDD-W) per study

	Burkina Faso	India	Malawi	Tanzania
Other fruits	• Watermelon<br>• Melon<br>•	• Bitter gourd<br>• Sponge gourd<br>• Ivy gourd<br>• Jackfruit<br>• Ash gourd<br>• Peer gourd<br>• Pointed gourd<br>• Ridge gourd<br>• Bottle gourd<br>• Snake gourd	• Mango<br>• Banana<br>• Citrus<br>• Guava<br>• Avocado	..
Cash Crops	• Cotton<br>• Hibiscus (bissap)	• Other cash crop<br>• Arum<br>• Coriander<br>• Other non-edible fibres<br>• Sugarcane<br>• Tobacco<br>• Garlic<br>• Cotton<br>• Ginger<br>• Turmeric	..	..

Table S4 - Assets included in Asset Score for each study

Burkina Faso*	India†	Malawi	Tanzania§
Agricultural land	House	Agricultural land	Land ownership
Large livestock	Large livestock	Large livestock	Metal Roof
Small livestock	Small livestock	Small livestock	Electricity
Fishpond	Mechanised farm equipment	Fishpond	Solar Panel
Non-mechanised farm equipment	Non-mechanised farm equipment	Farm equipment (mechanised)	Ox plow
Mechanised farm equipment	Business	Nonfarm business equipment	Cell Phone
House or building	High-cost consumer goods	House or other structures	Radio
Large consumer durable	Low-cost consumer goods	Cell Phone	Modern beds
Small consumer durable	Jewellery	Non-agricultural land	Mosquito net
Cell phone	Phone	Transport	Books
Nonagricultural land	Nonagricultural land	Checking	Bikes
Transportation	Agricultural land	Farming equipment (non-mechanised)	Cow
Concrete Floor	Improved toilet‡	Small consumer durables	
Iron Roof	Fuel	Household durables	
Banco Walls	Wall		
Improved Drinking Water Source‡	Roof		
Bed Nets	Floor		
Functional toilet			

*Calculated based on assets from male-reported version of the pro-WEAI and household survey.

† Calculated based on assets from male-reported version of the A-WEAI. When information was missing for non-agricultural land ownership, female responses were used.

‡ Improved Drinking Water Source defined as improved traditional well (covered), a tube well or borehole, protected well, rainwater, faucet/standpipe, and tanker truck. Improved toilet was categorised as flush to piped sewer system, septic tank, flush to pit latrine, ventilated improved latrine pit, pit latrine with slab, composting toilet, or not shared. All of these were based on DHS-7 criteria 2020.[26]

§ Information on assets came from household survey for Tanzania since that module was not collected through the A-WEAI.

Table S5 – Summary of incomplete information on women's empowerment in agriculture and cropping patterns for each study

Study	Incomplete information on Women's Empowerment	Incomplete information on cropping patterns	Incomplete information on both	Final Analytic Sample
**Burkina Faso**	38 (2·1)	27 (1·5)	0	1,735 (96·4)
**India**	24 (0·5)	7 (0·2)	1 (0·0)	4,450 (99·3)
**Malawi**	584 (46·5)	125 (10·0)	0	547 (43·6)
**Tanzania**	8 (1·3)	9 (1·5)	0	574 (97·1)

Values are number (percentage) referring to the number of participants and proportion of participants from the original sample with incomplete information.

Table S6 – Summary of sociodemographic characteristics across samples

	Burkina Faso (n = 1735)	India (n = 4465)	Malawi (n = 547)	Tanzania (n = 574)
**Mother's age (years)**				
Mean (SD)	33·4 (9.50)	24·5 (4·03)	32·2 (8·82)	30·2 (7·64)
**Mother's marital status**				
Married*	1683 (97·0%)	4418 (99·3%)	··	533 (92·9%)
Not married†	52 (3·0%)	32 (0.7%)	··	41 (7·1%)
**Polygynous**				
Yes	808 (46·6%)	··	19 (3·5%)	47 (8·2%)
**Education**				
No education	277 (16·0%)	1052 (23·6%)	248 (45·3%)	63 (11·0%)
At least some‡	1458 (84·0%)	3398 (76·4%)	299 (54·7%)	511 (89·0%)
**Household size**				
Mean (SD)	8.76 (4·65)	5.36 (2·05)	5·26 (1·71)	6.94 (2·21)
**Intervention assignment §**				
Control	880 (50·7%)	1053 (23·7%)	275 (50·3%)	288 (50·2%)
1	855 (49·3%)	1114 (25·0%)	272 (49·7%)	286 (49·8%)
2	··	1095 (24·6%)	··	··
3	··	1187 (26·7%)	··	··
**Household asset score¶**				
Mean (SD)	8.40 (1·77)	9·12 (2·63)	0·32 (0·14)	4·76 (1·91)
**Land size (ha)**				
Mean (SD)	··	1·68 (2·30)	··	5·23 (5·71)
Median [Min, Max]	··	1·10 [0, 62·7]	··	4·00 [0·10, 42·0]

* Burkina Faso “married” included: married living together/married living separately.
India “married” included: married. Tanzania “married” included: married/polygamous married.
†Burkina Faso included: never married/single/widow/separated/divorced/other. India “not married” included: separated/divorced/widowed/single.
Tanzania included: never married/separated/divorced/widowed.
‡ At least some education included either complete or incomplete formal education.
§ For Tanzania, Burkina Faso, and Malawi, second row refers to intervention group.
¶ The Asset Score was calculated as the sum of context-specific household assets; 17 assets for India, 12 for Tanzania, and 8 for Burkina Faso.
For Malawi, it was derived from 14 assets using Principal Component Analysis.
| | For India, recorded in Indian Rupee ₹ (INR). Conversion to United States Dollar is 1 dollar ≈ 79.90 INR.

Table S7 – Summary of women's empowerment count indicators across samples

	Burkina Faso (n = 1735)	India (n = 4450)	Malawi (n = 547)	Tanzania (n = 574)
**Input into productive decisions**				
**Number of areas women felt they could/had some input/decided**				
Mean (SD)	1·76 (0·855)	2·79 (1·21)	2·08 (1·10)	3·91 (1·23)
Median [Min, Max]	2·00 [0, 5·00]	3·00 [0, 5·00]	2·00 [0, 5·00]	4·00 [0, 7·00]
**Ownership of assets**				
**Number of small and large assets owned solely or jointly**				
Mean (SD)	3·03 (1·74)	3·91 (2·27)	..	..
Median [Min, Max]	3·00 [0, 10.0]	4·00 [0, 11·0]	..	..
**Number of small and large agricultural assets owned solely or jointly**				
Mean (SD)	1·56 (0·99)	1.21 (1.36)	..	..
Median [Min, Max]	1·00 [0, 6.00]	1·00 [0, 5·00]	..	..
**Access to and decisions on credit**				
**Number of credit sources she took out either solely or jointly * †**				
Mean (SD)	0·247 (0·57)	0·199 (0·48)	2·76 (1·64)	0·197 (0·42)
Median [Min, Max]	0 [0, 6·00]	0 [0, 3·00]	3·00 [1·00, 6·00]	0 [0, 2·00]
**Number of credit sources she solely made decisions to either borrow or use***				
Mean (SD)	0·127 (0·36)	0·0584 (0·262)	0.910 (1·00)	0 (0)
Median [Min, Max]	0 [0, 3·00]	0 [0, 3·00]	1·00 [0, 6·00]	0 [0, 0]
**Group membership**				
**Number of groups where she is an active member**				
Mean (SD)	0·475 (0·744)	0·409 (0·75)	1·16 (1·18)	0·232 (0·645)
Median [Min, Max]	0 [0, 4·00]	0 [0, 6·00]	1·00 [0, 6·00]	0 [0, 6·00]
**Workload**				
**Number of productive hours in the previous 24 hours**				
Mean (SD)	6·68 (3·29)	20·7 (3·57)	11·9 (4·06)	9·58 (2·79)
Median [Min, Max]	6·69 [0, 22·3]	22·0 [0, 24·0]	11·5 [2·00, 24·0]	10·0 [1·00, 19·0]

*Given that household has access to that form of credit/loan.
† For India, this is used as a proxy for household access to credit since information on access was not available.

Table S8 – Summary of women's empowerment binary indicators across samples

	Burkina Faso (n = 1735)	India (n = 4450)	Malawi (n = 547)	Tanzania (n = 574)
**Input into productive decisions**				
**Women felt they could/had some input/decided in 2 or more areas**				
Empowered	1149 (66·2%)	3691 (82·9%)	355 (64·9%)	540 (94·1%)
**Ownership of assets**				
**Owns at least two small assets or one large asset***				
Empowered	1596 (92·0%)	4062 (91·3%)	..	..
**Access to and decisions on credit**				
**Has at least one form of credit or makes decisions on at least one type of credit†**				
Empowered	368 (21·2%)	753 (16·9%)	353 (64·5%)	109 (19·0%)
**Group membership**				
**Active member in at least one group**				
Empowered	603 (34·8%)	1333 (30·0%)	361 (66·0%)	92 (16·0%)
**Workload**				
**Woman worked <10.5 productive hours in the previous 24 hours**				
Empowered	1507 (86·9%)	1787 (40·0%)	196 (35·8%)	320 (55·7%)

*Small assets consisted of small livestock, non-mechanised farm equipment, or low-cost durables. Large assets consisted of large livestock, agricultural land ownership, mechanised farm equipment, non-farm commercial equipment, house or building, high-cost durables, cell phone, non-agricultural land, or method of transport.

†Adequate if using at least one source of credit or made decision solely regarding at least one source of credit given that household has access. Credit sources vary according to country **Table S1.**

Table S9 - Summary of workload indicators including childcare across samples

	Burkina Faso (n = 1735)	India (n = 4450)	Malawi (n = 547)	Tanzania (n = 574)
**Works less than 10.5 hours/day including childcare**				
Empowered	694 (40·0%)	111 (2·5%)	..	116 (20·2%)
**Number of productive hours including childcare**				
Mean (SD)	12.2 (5·41)	15·8 (2·74)	..	11.8 (2·14)
Median [Min, Max]	12·5 [0, 24·0]	15·8 [1·00, 24·0]	..	12·0 [1·00, 19·3]

Figure S2 – Cultivation of food groups by country

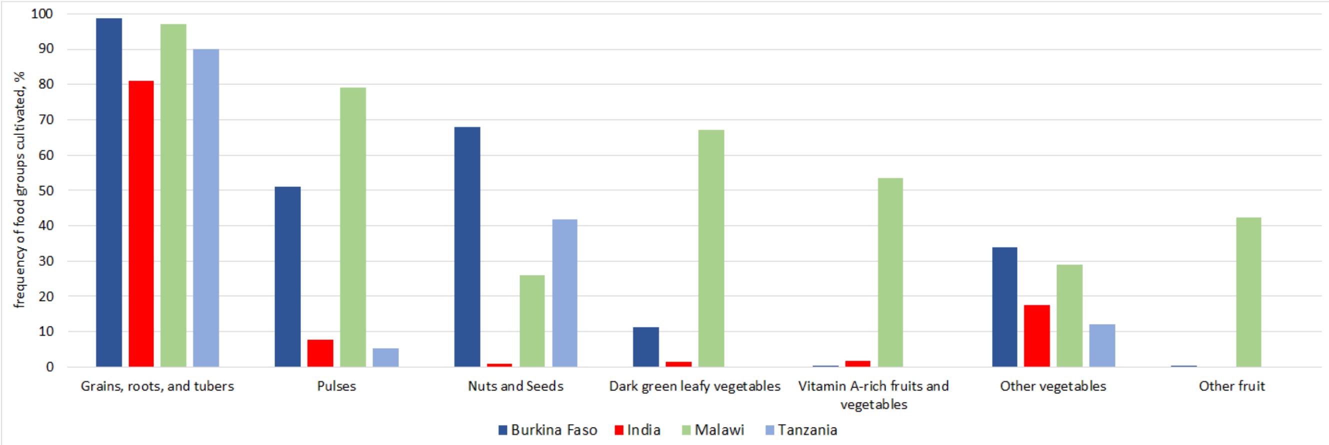

Sample sizes are as follows: Burkina Faso (n=1735), India (n=4450), Malawi (n=547), and Tanzania (n=574). Food groups refer to food group categories. Food group names are abbreviated and are as follows: 'Grains, roots, and tubers' includes grains, white roots and tubers and plantains; 'Pulses' includes beans, peas, and lentils. See **table S2** for specific food items within each food group category by country.

Figure S3 – Crop diversity by country

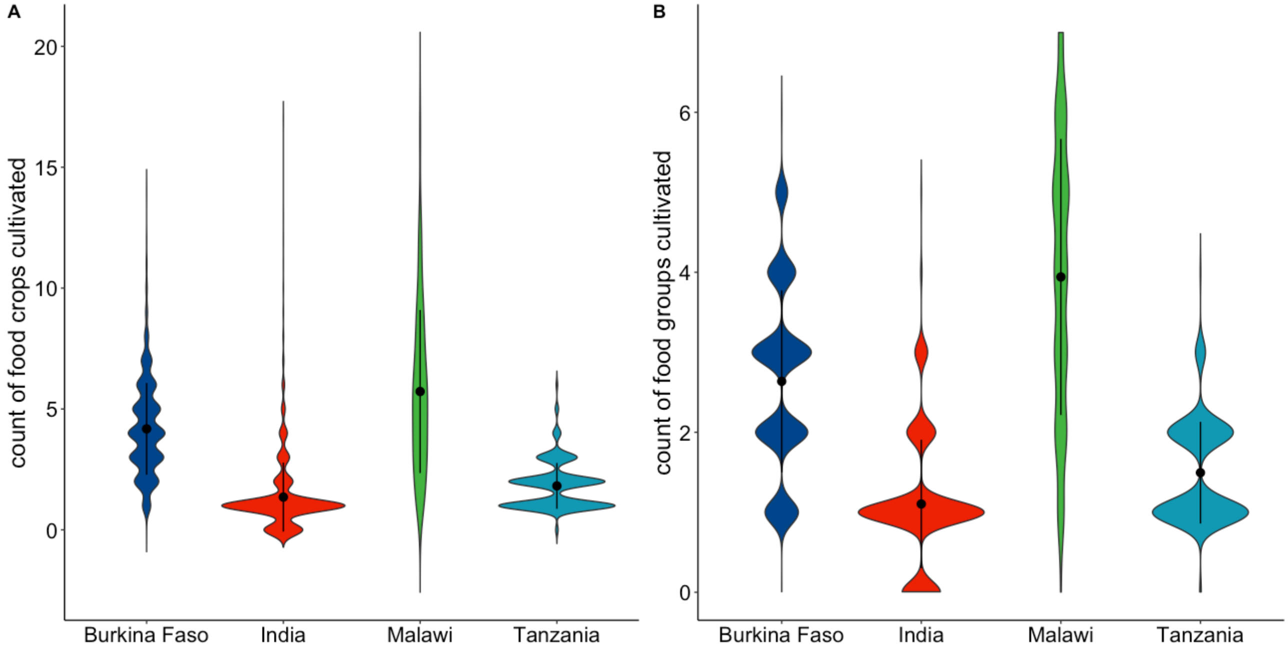

A corresponds to total food crop diversity and **B** corresponds to total food group diversity (range: 0 – 7 food groups). Above are violin plots. The centre black dot represents the sample average, whereas the centre line represents the interquartile range. The full plot illustrates the full range, and the body indicates the distribution of the data. Bulges represent clustering of responses around that point. The mean (standard deviation) for food crop count by country are as follows: Burkina Faso 4·18 (1·90); India 1·36 (1·43); Malawi 5·72 (3·37); Tanzania 1·82 (0·95). The mean (standard deviation) for food group count by country are as follows: Burkina Faso 2·64 (1·14); India 1·10 (0·80); Malawi 3·94 (1·73); Tanzania 1·49 (0·64).

Figure S4 – Input into productive decisions and crop diversity

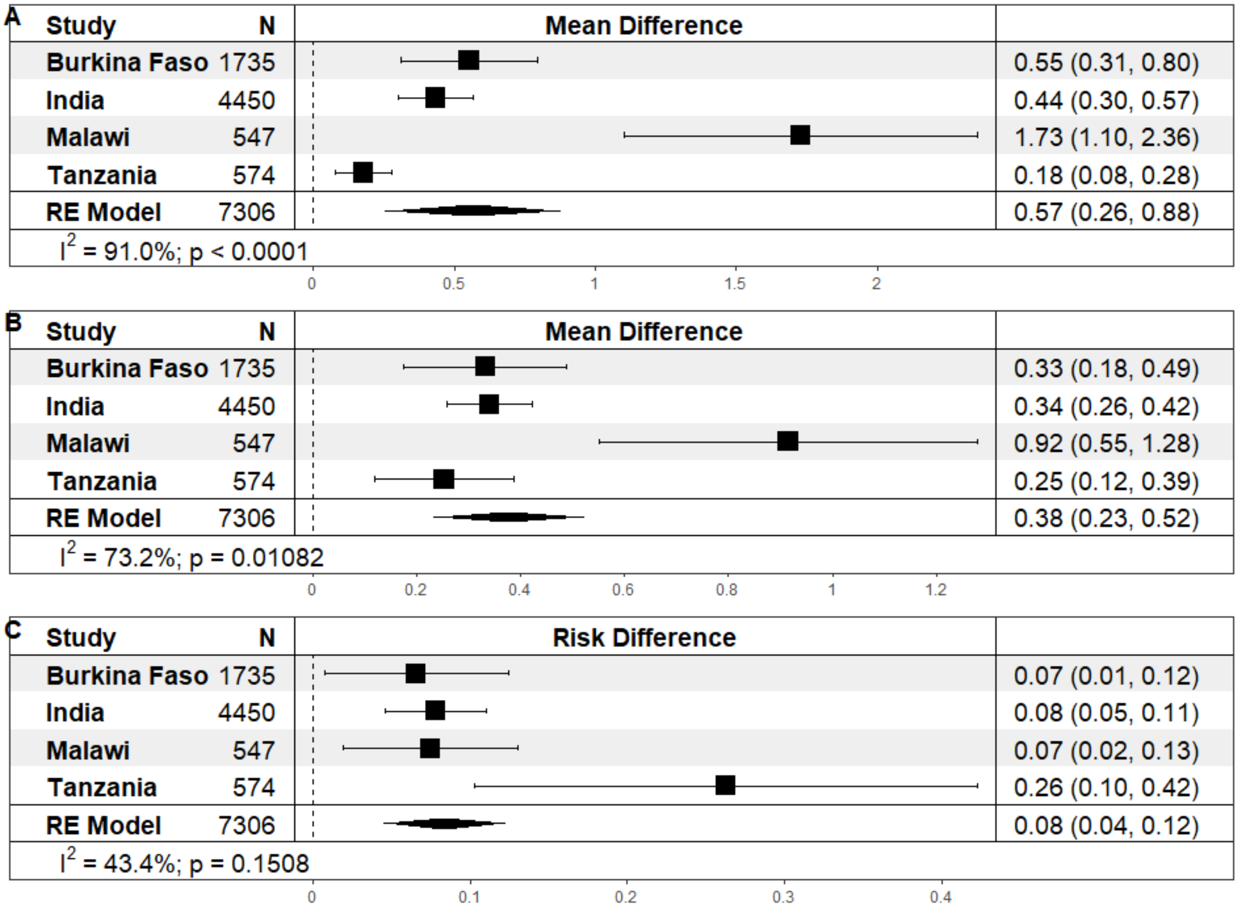

A corresponds to total food crop diversity, **B** corresponds to total food group diversity (range: 0 – 7 food groups), and **C** corresponds to cultivation of nutrient dense crops (defined as such if they reported cultivating at least one crop in the following food group categories: pulses, nuts and seeds, dark green leafy vegetables, other vitamin-A rich fruits and vegetables, other vegetables, or other fruit. Non-cultivators of nutrient dense crops were those who grew only grains, roots, and tubers or if they grew no food groups (meaning not growing anything at all or only growing cash crops).

Input into productive decisions was defined as empowered if has some input or input into most or all decisions or made the decision in two or more areas.

We pooled country-specific adjusted estimates using a random effects model using the DerSimonian and Laird method.

The adjusted model for Burkina Faso controlled for intervention assignment, age, educational attainment, asset score, household size, and polygyny. The model for India controlled for intervention assignment, age, educational attainment, asset score, household size, and land size. The model for Malawi controlled for intervention assignment, age, educational attainment, asset score, and household size. The model for Tanzania controlled for intervention assignment, age, educational attainment, asset score, household size, and land size.

Figure S5 – Ownership of assets and crop diversity

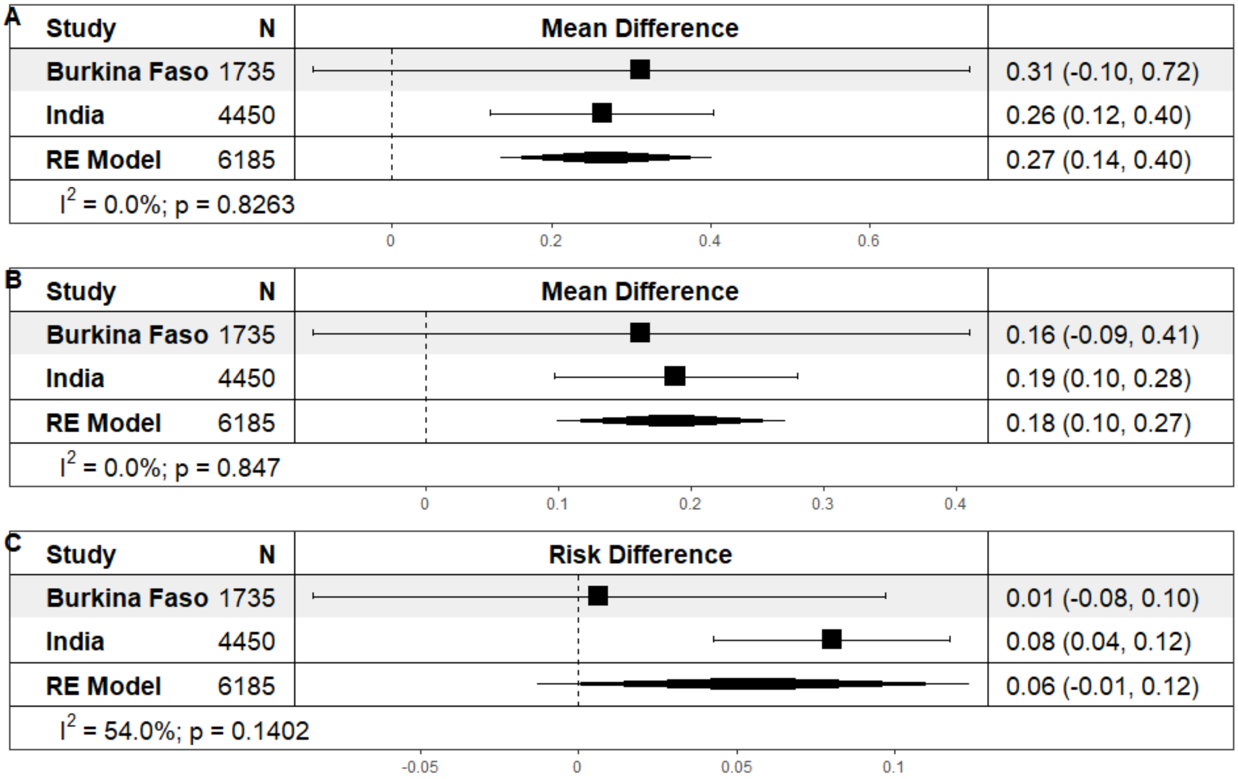

A corresponds to total food crop diversity, **B** corresponds to total food group diversity (range: 0 – 7 food groups), and **C** corresponds to cultivation of nutrient dense crops (defined as such if they reported cultivating at least one crop in the following food group categories: pulses, nuts and seeds, dark green leafy vegetables, other vitamin-A rich fruits and vegetables, other vegetables, or other fruit. Non-cultivators of nutrient dense crops were those who grew only grains, roots, and tubers or if they grew no food groups (meaning not growing anything at all or only growing cash crops).

Ownership of assets was defined as empowered if owned at least one large asset or at least two small assets.

We pooled country-specific adjusted estimates using a random effects model using the DerSimonian and Laird method.

The adjusted model for Burkina Faso controlled for intervention assignment, age, educational attainment, household size, and polygyny. The model for India controlled for intervention assignment, age, educational attainment, household size, and land size. The model for Malawi controlled for intervention assignment, age, educational attainment, and household size. The model for Tanzania controlled for intervention assignment, age, educational attainment, household size, and land size.

Figure S6 – Access to and decisions on credit and crop diversity

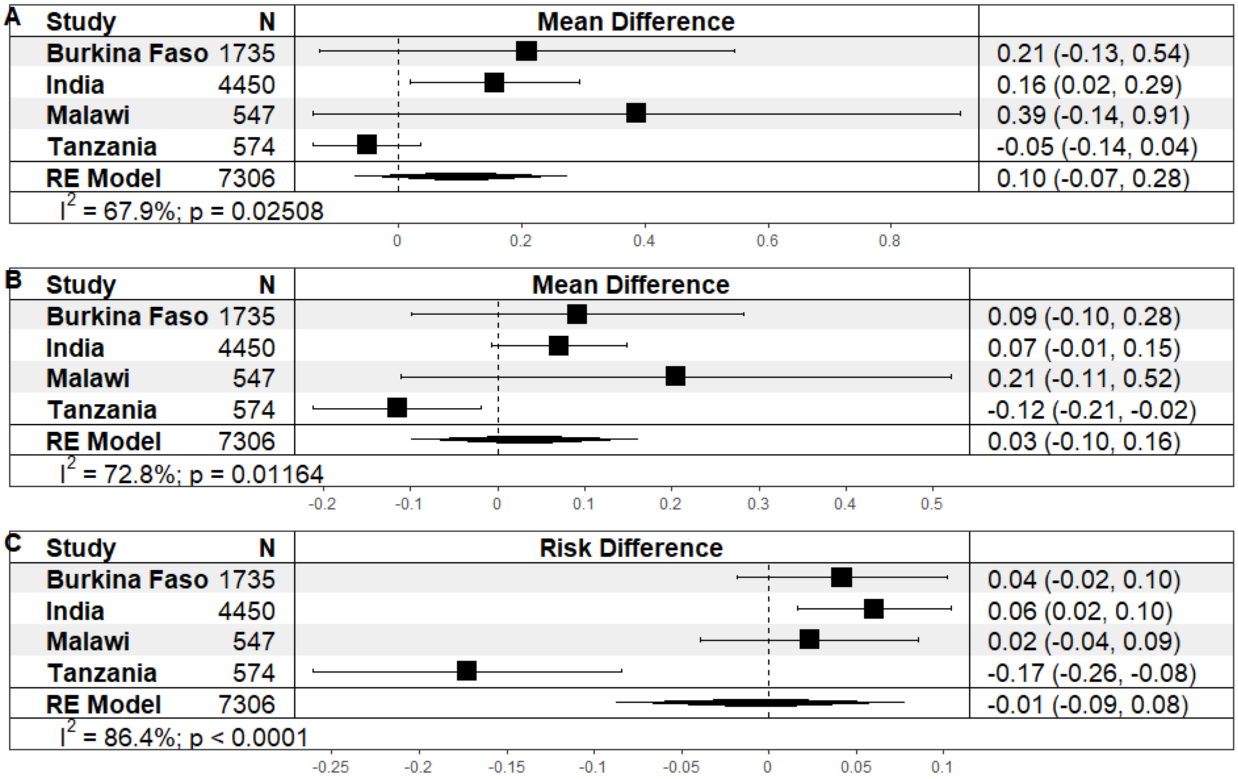

A corresponds to total food crop diversity, **B** corresponds to total food group diversity (range: 0 – 7 food groups), and **C** corresponds to cultivation of nutrient dense crops (defined as such if they reported cultivating at least one crop in the following food group categories: pulses, nuts and seeds, dark green leafy vegetables, other vitamin-A rich fruits and vegetables, other vegetables, or other fruit. Non-cultivators of nutrient dense crops were those who grew only grains, roots, and tubers or if they grew no food groups (meaning not growing anything at all or only growing cash crops).

Access to and decisions on credit was defined as empowered if using at least one source of credit or made decision solely regarding at least one source of credit given that household has access.

We pooled country-specific adjusted estimates using a random effects model using the DerSimonian and Laird method.

The adjusted model for Burkina Faso controlled for intervention assignment, age, educational attainment, asset score, household size, polygyny, and active membership of a credit/microfinance lending group. The model for India controlled for intervention assignment, age, educational attainment, asset score, household size, land size, and active membership of a credit/microfinance lending group. The model for Malawi controlled for intervention assignment, age, educational attainment, asset score, household size, and active membership of a credit/microfinance lending group. The model for Tanzania controlled for intervention assignment, age, educational attainment, asset score, household size, land size, and active membership of a credit/microfinance lending group.

Figure S7 – Group membership and crop diversity

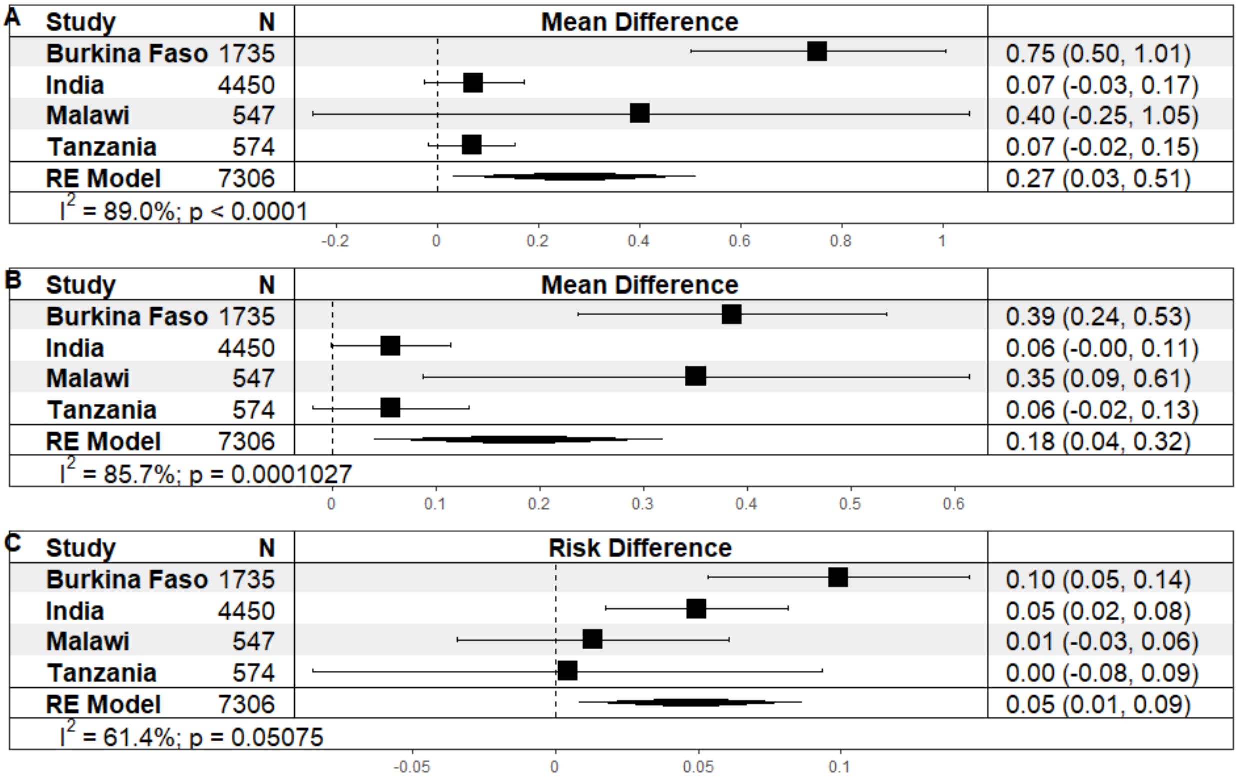

A corresponds to total food crop diversity, **B** corresponds to total food group diversity (range: 0 – 7 food groups), and **C** corresponds to cultivation of nutrient dense crops (defined as such if they reported cultivating at least one crop in the following food group categories: pulses, nuts and seeds, dark green leafy vegetables, other vitamin-A rich fruits and vegetables, other vegetables, or other fruit. Non-cultivators of nutrient dense crops were those who grew only grains, roots, and tubers or if they grew no food groups (meaning not growing anything at all or only growing cash crops).

Group membership was defined as empowered if active member in at least one group.

We pooled country-specific adjusted estimates using a random effects model using the DerSimonian and Laird method.

The adjusted model for Burkina Faso controlled for intervention assignment, age, educational attainment, asset score, household size, and polygyny. The model for India controlled for intervention assignment, age, educational attainment, asset score, household size, and land size. The model for Malawi controlled for intervention assignment, age, educational attainment, asset score, and household size. The model for Tanzania controlled for intervention assignment, age, educational attainment, asset score, household size, and land size.

Figure S8 – Work balance and crop diversity

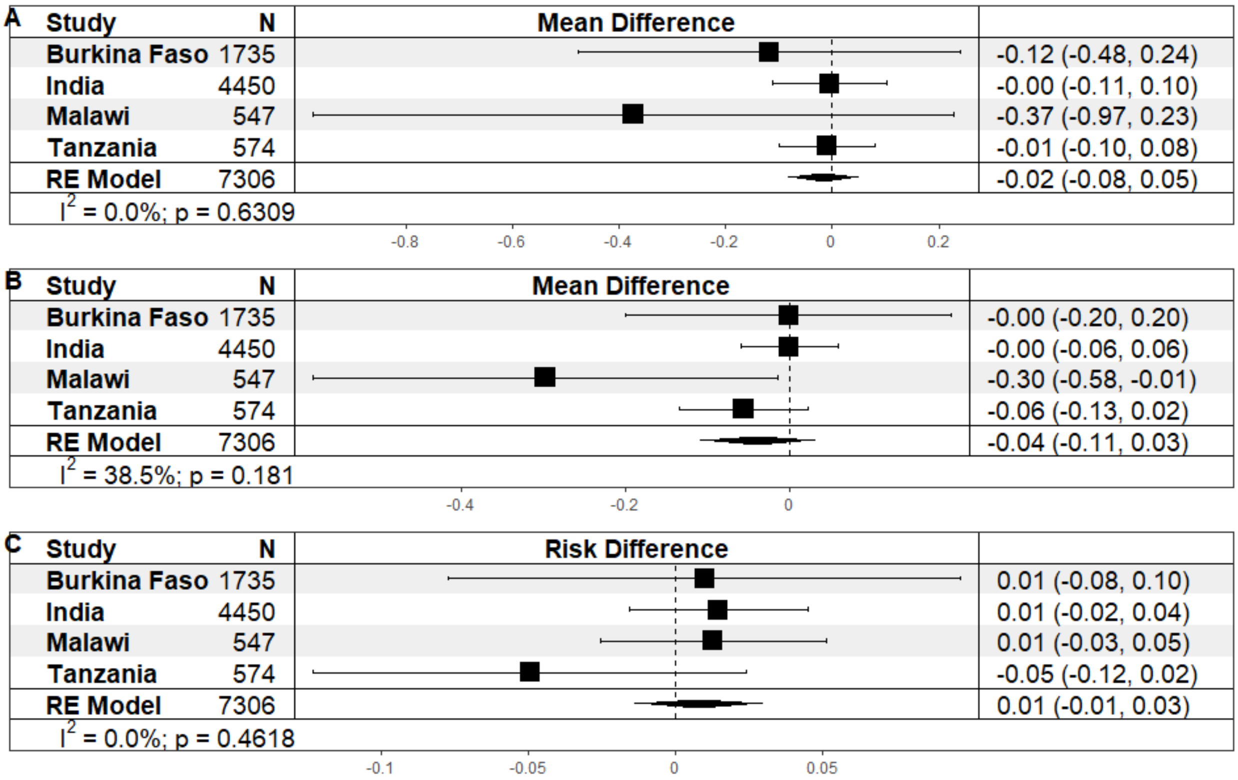

A corresponds to total food crop diversity, **B** corresponds to total food group diversity (range: 0 – 7 food groups), and **C** corresponds to cultivation of nutrient dense crops (defined as such if they reported cultivating at least one crop in the following food group categories: pulses, nuts and seeds, dark green leafy vegetables, other vitamin-A rich fruits and vegetables, other vegetables, or other fruit. Non-cultivators of nutrient dense crops were those who grew only grains, roots, and tubers or if they grew no food groups (meaning not growing anything at all or only growing cash crops).

Work balance was defined as empowered if works less than 10.5 productive hours in one day. This does not consider time dedicated to childcare.

We pooled country-specific adjusted estimates using a random effects model using the DerSimonian and Laird method.

The adjusted model for Burkina Faso controlled for intervention assignment, age, educational attainment, asset score, household size, and polygyny. The model for India controlled for intervention assignment, age, educational attainment, asset score, household size, and land size. The model for Malawi controlled for intervention assignment, age, educational attainment, asset score, and household size. The model for Tanzania controlled for intervention assignment, age, educational attainment, asset score, household size, and land size.

Figure S9 – Productive work hours including childcare and crop diversity

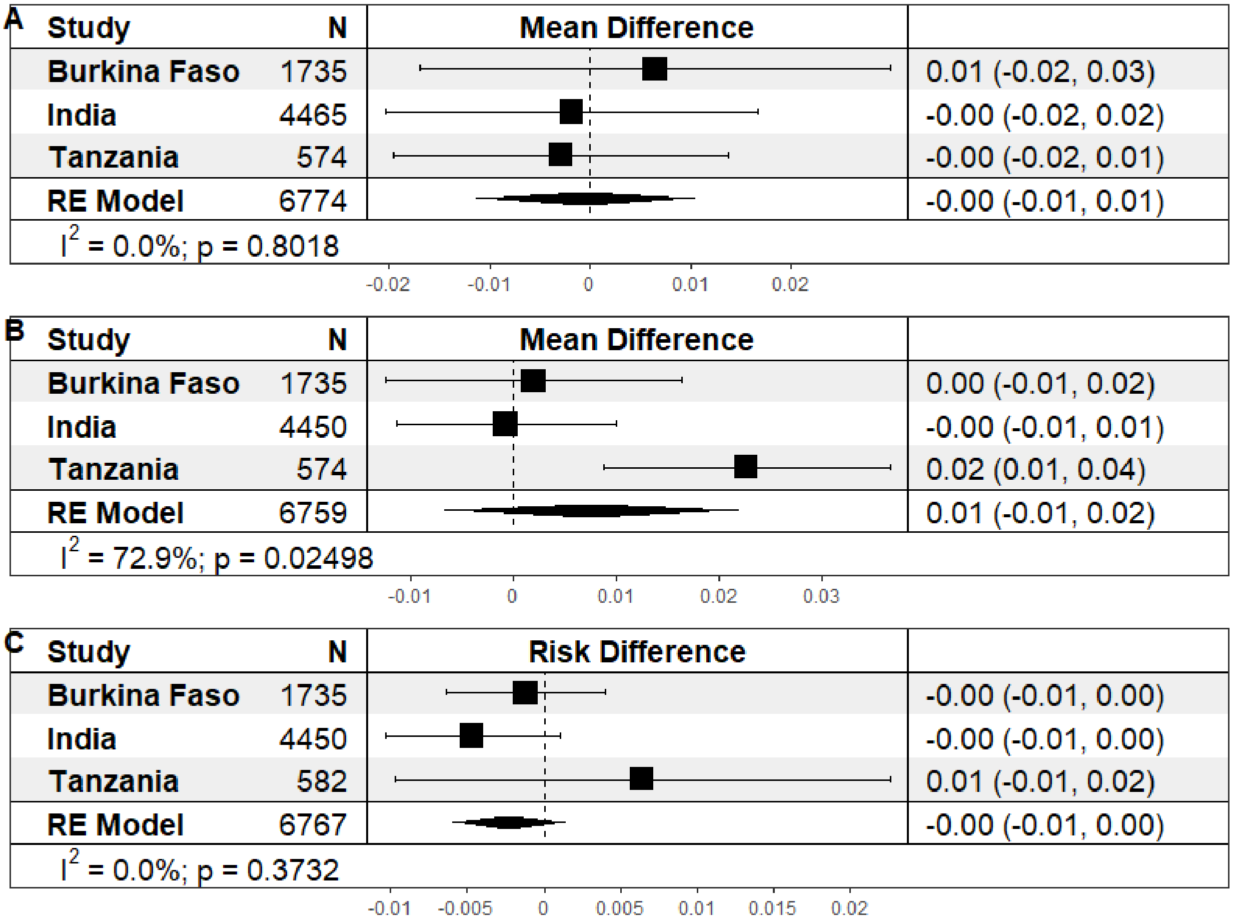

A corresponds to total food crop diversity, **B** corresponds to total food group diversity (range: 0 – 7 food groups), and **C** corresponds to cultivation of nutrient dense crops (defined as such if they reported cultivating at least one crop in the following food group categories: pulses, nuts and seeds, dark green leafy vegetables, other vitamin-A rich fruits and vegetables, other vegetables, or other fruit. Non-cultivators of nutrient dense crops were those who grew only grains, roots, and tubers or if they grew no food groups (meaning not growing anything at all or only growing cash crops).
Productive work hours were defined as the number of hours dedicated to productive work per day per individual, including childcare.
We pooled country-specific adjusted estimates using a random effects model using the DerSimonian and Laird method.
The adjusted model for Burkina Faso controlled for intervention assignment, age, educational attainment, asset score, household size, and polygyny. The model for India controlled for intervention assignment, age, educational attainment, asset score, household size, and land size. The model for Malawi controlled for intervention assignment, age, educational attainment, asset score, and household size. The model for Tanzania controlled for intervention assignment, age, educational attainment, asset score, household size, and land size.

Figure S10 – Work balance including childcare and crop diversity

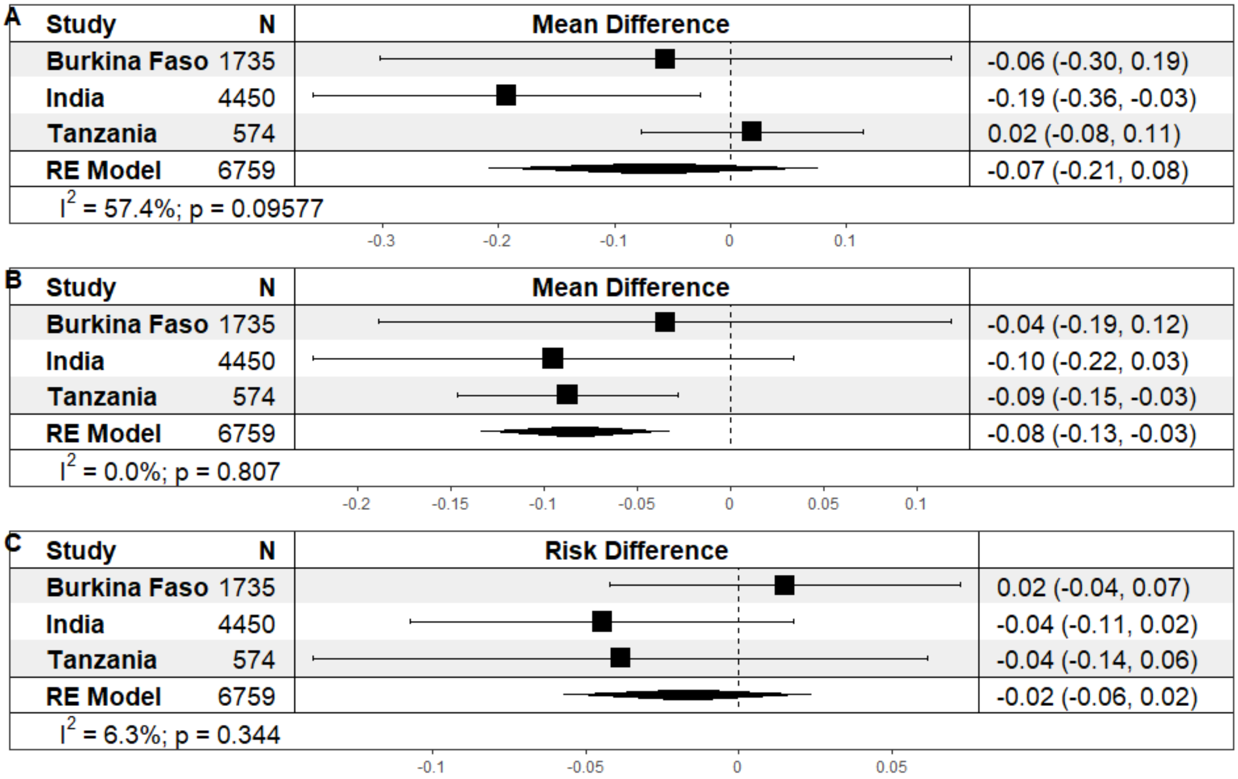

A corresponds to total food crop diversity, **B** corresponds to total food group diversity (range: 0 – 7 food groups), and **C** corresponds to cultivation of nutrient dense crops (defined as such if they reported cultivating at least one crop in the following food group categories: pulses, nuts and seeds, dark green leafy vegetables, other vitamin-A rich fruits and vegetables, other vegetables, or other fruit. Non-cultivators of nutrient dense crops were those who grew only grains, roots, and tubers or if they grew no food groups (meaning not growing anything at all or only growing cash crops).

Work balance was defined as empowered if works less than 10·5 productive hours in one day, including childcare.

We pooled country-specific adjusted estimates using a random effects model using the DerSimonian and Laird method.

The adjusted model for Burkina Faso controlled for intervention assignment, age, educational attainment, asset score, household size, and polygyny. The model for India controlled for intervention assignment, age, educational attainment, asset score, household size, and land size. The model for Malawi controlled for intervention assignment, age, educational attainment, asset score, and household size. The model for Tanzania controlled for intervention assignment, age, educational attainment, asset score, household size, and land size.

Figure S11 –Input into productive decisions and crop diversity

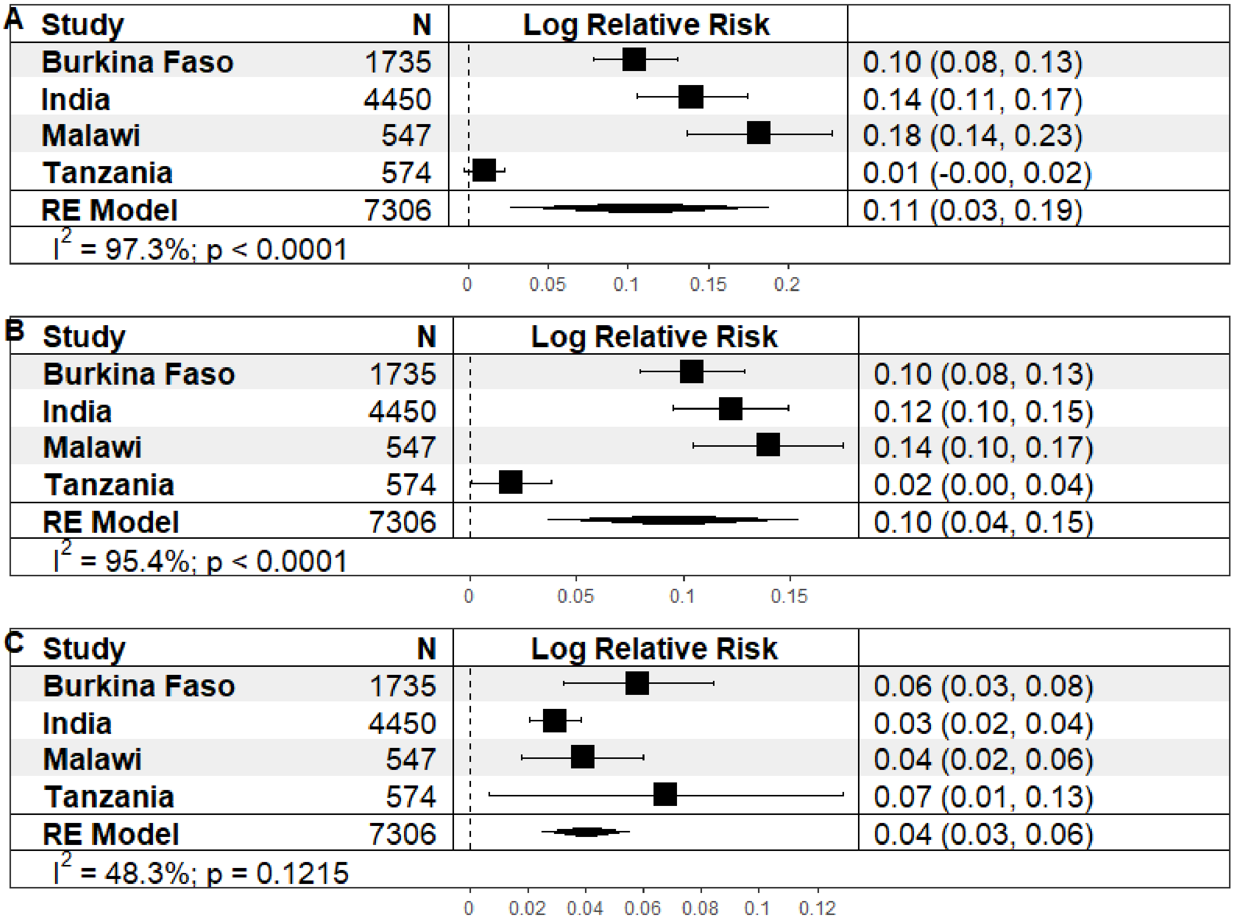

A corresponds to total food crop diversity, **B** corresponds to total food group diversity (range: 0 – 7 food groups), and **C** corresponds to cultivation of nutrient-dense crops (defined as cultivating at least one crop in the following food group categories: pulses, nuts and seeds, dark green leafy vegetables, other vitamin-A rich fruits and vegetables, other vegetables, or other fruit. Non-cultivators of nutrient -dense crops were those who grew only grains, roots, and tubers or if they grew no food groups (meaning not growing anything at all or only growing cash crops).

Input into productive decisions was defined as the number of areas the woman had some input in decisions or felt she could make decisions or made decisions herself.

We pooled country-specific adjusted estimates using a random effects model using the DerSimonian and Laird method.

The adjusted model for Burkina Faso controlled for intervention assignment, age, educational attainment, asset score, household size, and polygyny. The model for India controlled for intervention assignment, age, educational attainment, asset score, household size, and land size. The model for Malawi controlled for intervention assignment, age, educational attainment, asset score, and household size. The model for Tanzania controlled for intervention assignment, age, educational attainment, asset score, household size, and land size.

Figure S12 – Ownership of Assets and Crop Diversity

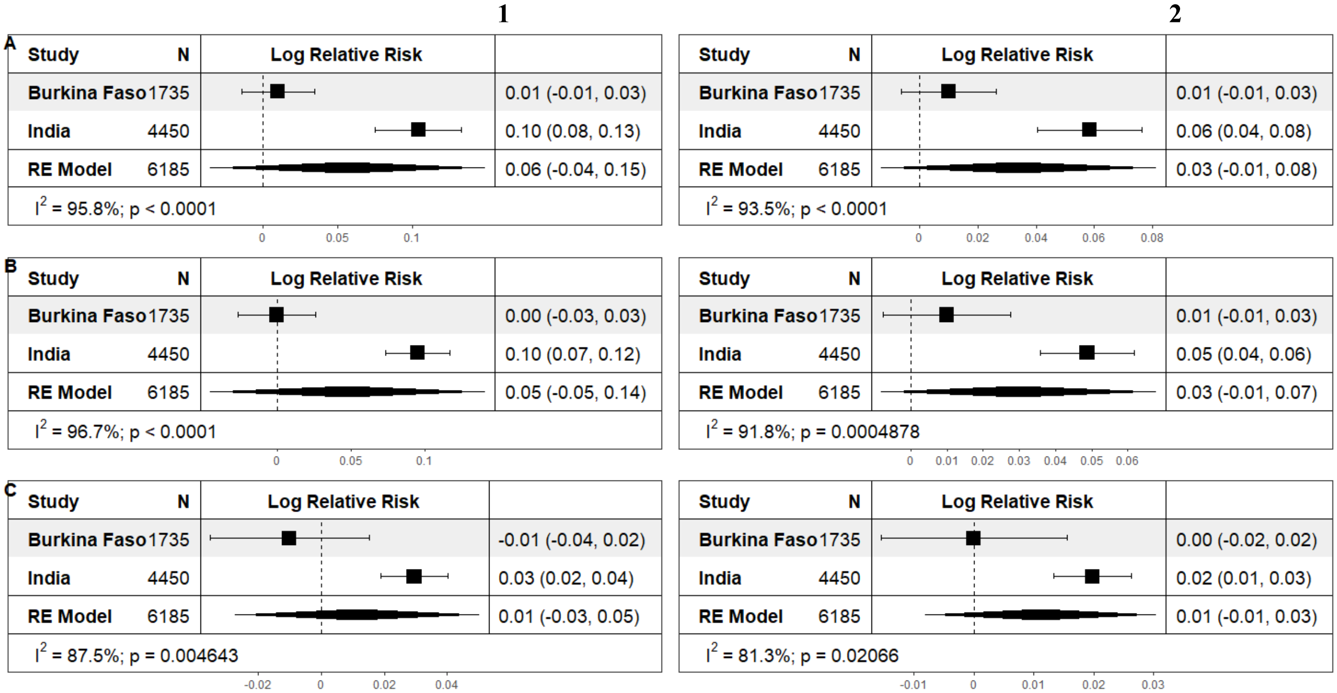

Columns: **1** refers to agricultural assets defined as the number of agricultural assets that the woman owned herself or jointly. **2** refers to assets defined as the number of assets (including agricultural assets) that the woman owned herself or jointly.

Rows: **A** corresponds to total food crop diversity, **B** corresponds to total food group diversity (range: 0 – 7 food groups), and **C** corresponds to cultivation of nutrient-dense crops (defined as cultivating at least one crop in the following food group categories: pulses, nuts and seeds, dark green leafy vegetables, other vitamin-A rich fruits and vegetables, other vegetables, or other fruit. Non-cultivators of nutrient -dense crops were those who grew only grains, roots, and tubers or if they grew no food groups (meaning not growing anything at all or only growing cash crops).

We pooled country-specific adjusted estimates using a random effects model using the DerSimonian and Laird method.

The adjusted model for Burkina Faso controlled for intervention assignment, age, educational attainment, household size, and polygyny. The model for India controlled for intervention assignment, age, educational attainment, household size, and land size. The model for Malawi controlled for intervention assignment, age, educational attainment, and household size. The model for Tanzania controlled for intervention assignment, age, educational attainment, household size, and land size.

Figure S13 – Access to and Decisions on Credit and Crop Diversity

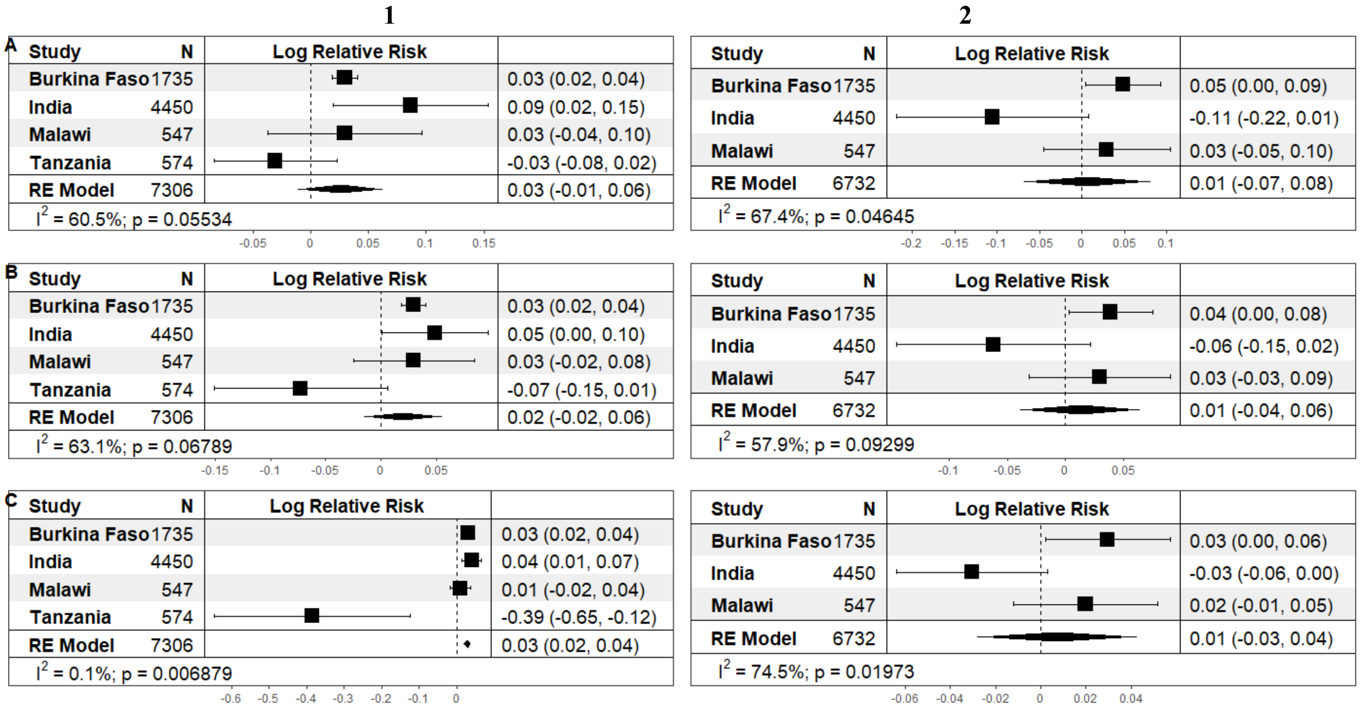

Column: **1** refers to credit use defined as the number of sources of credit that anyone in the woman's household (including herself) had borrowed/used in the past 12 months. **2** refers to decisions on credit defined as the number of sources of credit that the woman solely made the decision to either borrow from or how to use it in the last 12 months given that their household has access to that source of credit. Tanzania is not included in this meta-analysis due to zero women reporting making decisions on credit.

Rows: **A** corresponds to total food crop diversity, **B** corresponds to total food group diversity (range: 0 – 7 food groups), and **C** corresponds to cultivation of nutrient -dense crops (defined as cultivating at least one crop in the following food group categories: pulses, nuts and seeds, dark green leafy vegetables, other vitamin-A rich fruits and vegetables, other vegetables, or other fruit. Non-cultivators of nutrient -dense crops were those who grew only grains, roots, and tubers or if they grew no food groups (meaning not growing anything at all or only growing cash crops).

Credit use was defined as the number of sources of credit that anyone in the woman's household (including herself) had borrowed/used in the past 12 months.

We pooled country-specific adjusted estimates using a random effects model using the DerSimonian and Laird method.

The adjusted model for Burkina Faso controlled for intervention assignment, age, educational attainment, asset score, household size, polygyny, and active membership of a credit/microfinance lending group. The model for India controlled for intervention assignment, age, educational attainment, asset score, household size, land size, and active membership of a credit/microfinance lending group. The model for Malawi controlled for intervention assignment, age, educational attainment, asset score, household size, and active membership of a credit/microfinance lending group. The model for Tanzania controlled for intervention assignment, age, educational attainment, asset score, household size, land size, and active membership of a credit/microfinance lending group.

Figure S14 – Group Membership and Crop Diversity

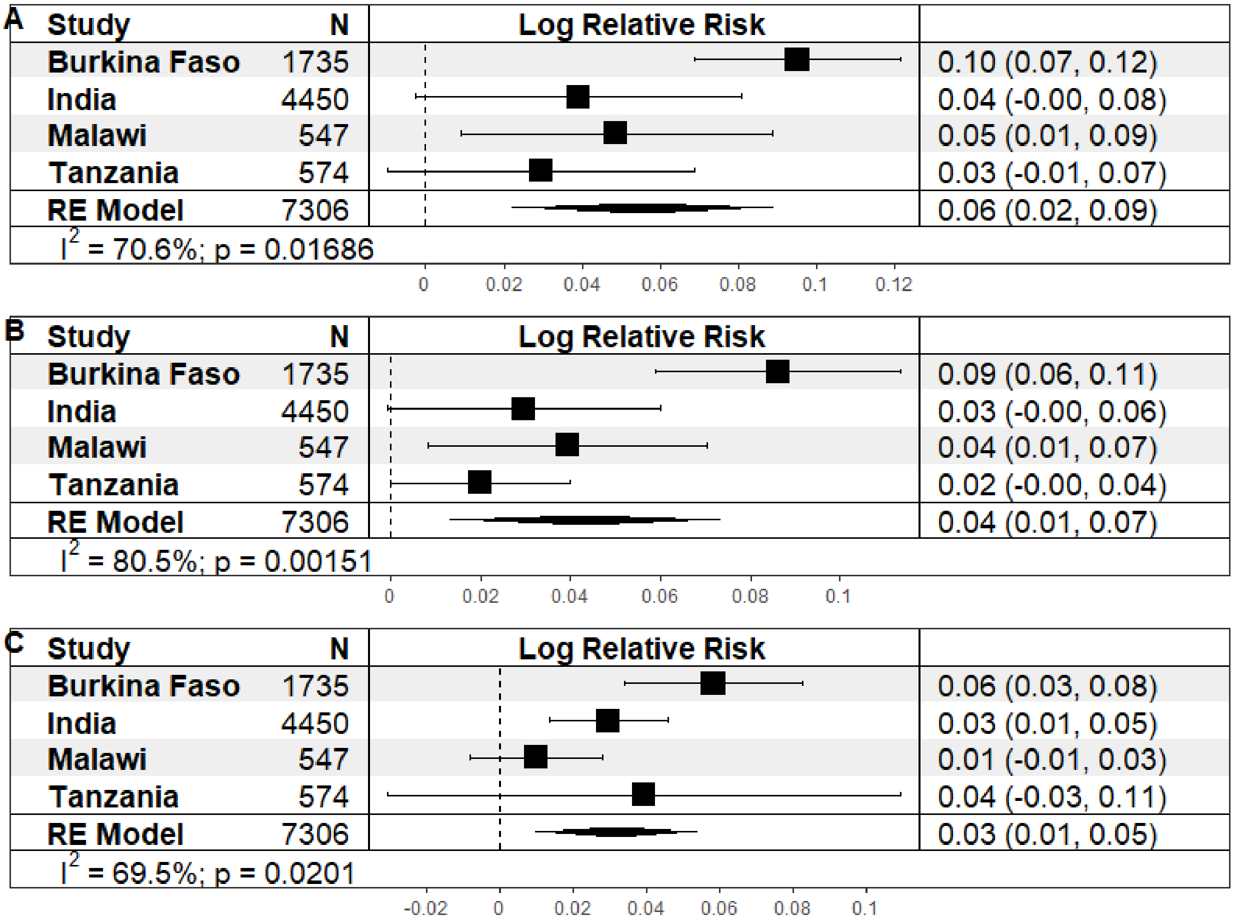

A corresponds to total food crop diversity, **B** corresponds to total food group diversity (range: 0 – 7 food groups), and **C** corresponds to cultivation of nutrient-dense crops (defined cultivating at least one crop in the following food group categories: pulses, nuts and seeds, dark green leafy vegetables, other vitamin-A rich fruits and vegetables, other vegetables, or other fruit. Non-cultivators of nutrient -dense crops were those who grew only grains, roots, and tubers or if they grew no food groups (meaning not growing anything at all or only growing cash crops).
Group membership was defined as the number of groups a woman was an active member of. If the woman reported no groups in their community, they were considered a member of zero groups.
We pooled country-specific adjusted estimates using a random effects model using the DerSimonian and Laird method.
The adjusted model for Burkina Faso controlled for intervention assignment, age, educational attainment, asset score, household size, and polygyny. The model for India controlled for intervention assignment, age, educational attainment, asset score, household size, and land size. The model for Malawi controlled for intervention assignment, age, educational attainment, asset score, and household size. The model for Tanzania controlled for intervention assignment, age, educational attainment, asset score, household size, and land size.

Figure S15 – Productive Work Hours and Crop Diversity

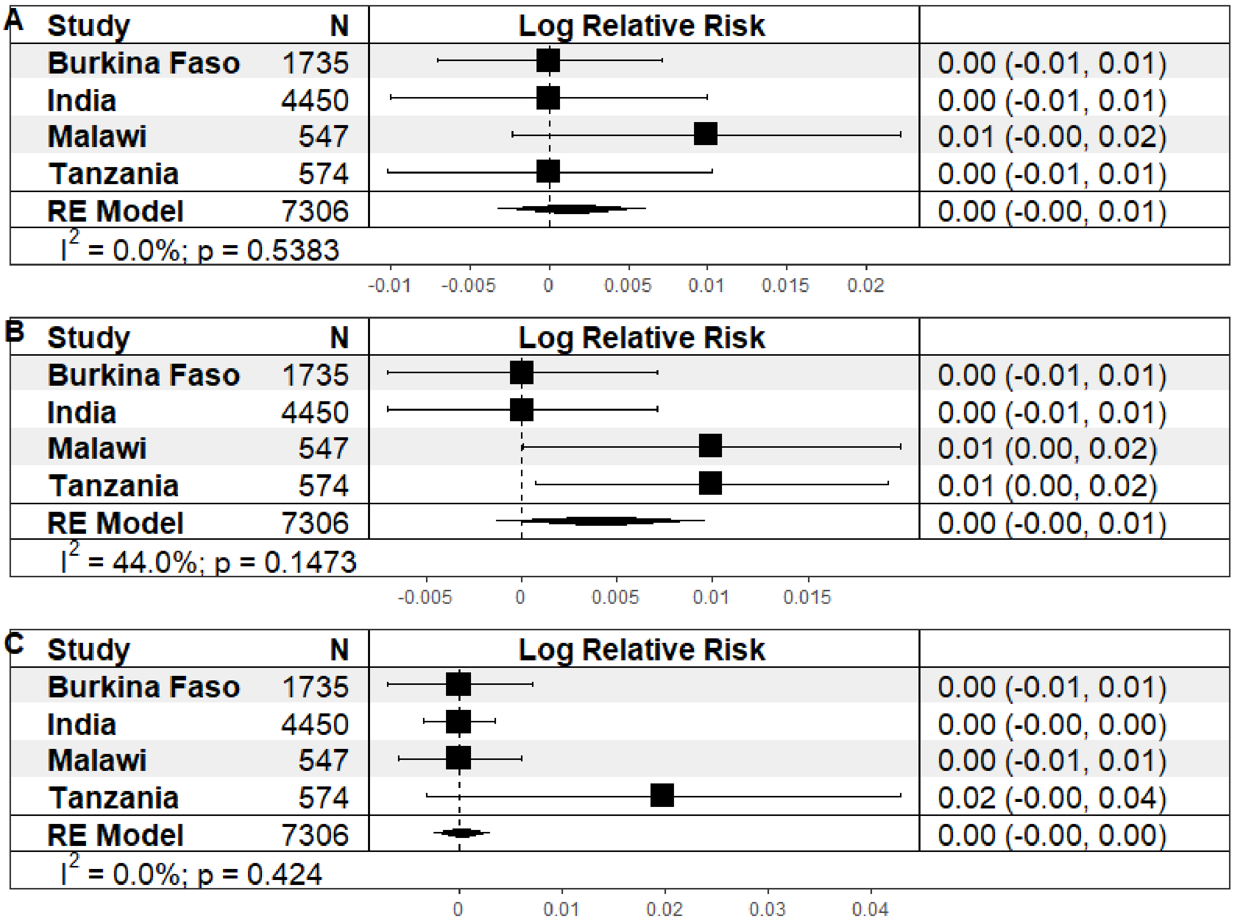

A corresponds to total food crop diversity, **B** corresponds to total food group diversity (range: 0 – 7 food groups), and **C** corresponds to cultivation of nutrient-dense crops (defined cultivating at least one crop in the following food group categories: pulses, nuts and seeds, dark green leafy vegetables, other vitamin-A rich fruits and vegetables, other vegetables, or other fruit. Non-cultivators of nutrient-dense crops were those who grew only grains, roots, and tubers or if they grew no food groups (meaning not growing anything at all or only growing cash crops).

Productive work hours were defined as the number of hours dedicated to productive work per day per individual. This version of the indicator does not include childcare.

We pooled country-specific adjusted estimates using a random effects model using the DerSimonian and Laird method.

The adjusted model for Burkina Faso controlled for intervention assignment, age, educational attainment, asset score, household size, and polygyny. The model for India controlled for intervention assignment, age, educational attainment, asset score, household size, and land size. The model for Malawi controlled for intervention assignment, age, educational attainment, asset score, and household size. The model for Tanzania controlled for intervention assignment, age, educational attainment, asset score, household size, and land size.

Figure S16 – Input into productive decisions and crop diversity

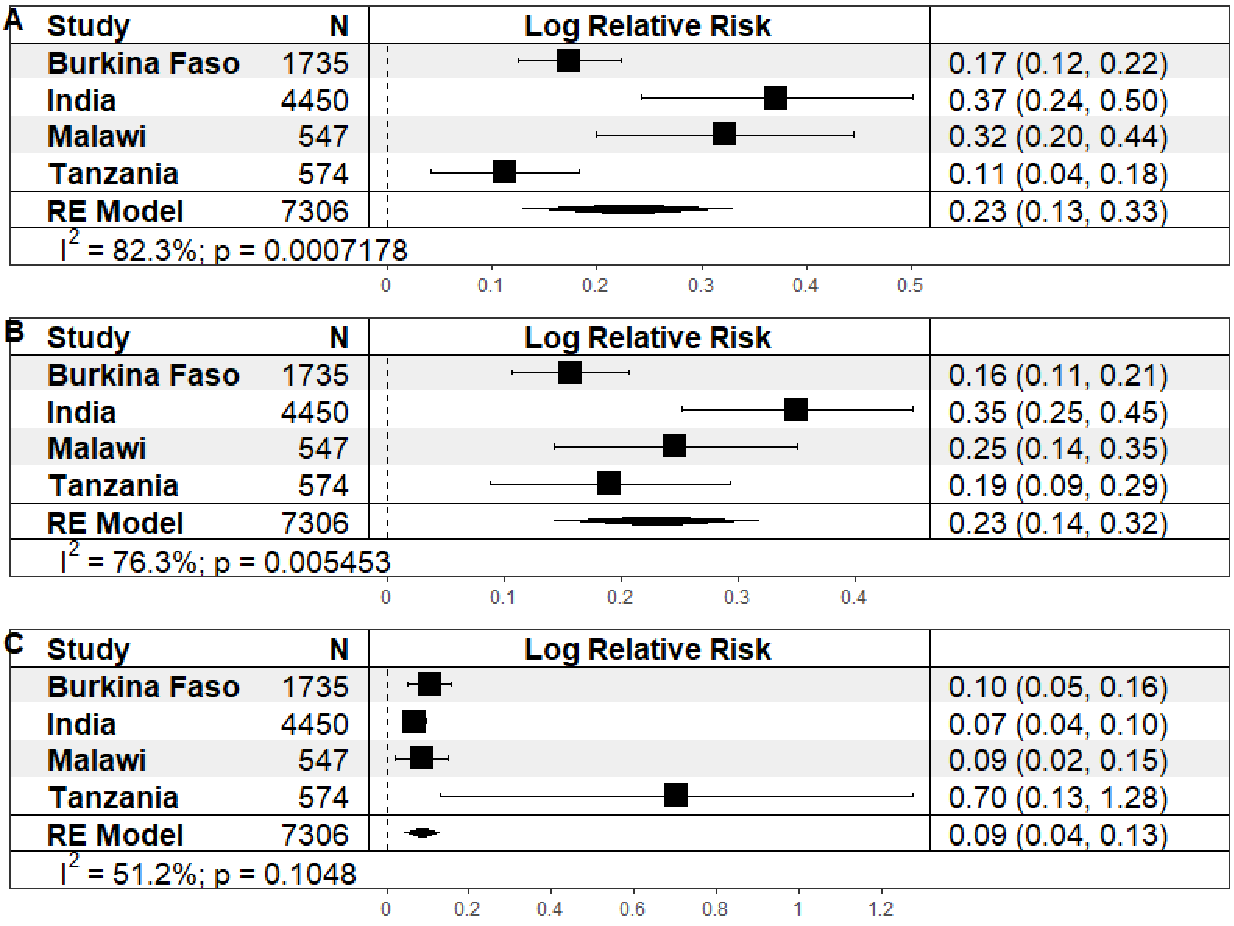

A corresponds to total food crop diversity, **B** corresponds to total food group diversity (range: 0 – 7 food groups), and **C** corresponds to cultivation of nutrient dense crops (defined as such if they reported cultivating at least one crop in the following food group categories: pulses, nuts and seeds, dark green leafy vegetables, other vitamin-A rich fruits and vegetables, other vegetables, or other fruit. Non-cultivators of nutrient dense crops were those who grew only grains, roots, and tubers or if they grew no food groups (meaning not growing anything at all or only growing cash crops).

Input into productive decisions was defined as empowered if has some input or input into most or all decisions or made the decision in two or more areas.

We pooled country-specific adjusted estimates using a random effects model using the DerSimonian and Laird method.

The adjusted model for Burkina Faso controlled for intervention assignment, age, educational attainment, asset score, household size, and polygyny. The model for India controlled for intervention assignment, age, educational attainment, asset score, household size, and land size. The model for Malawi controlled for intervention assignment, age, educational attainment, asset score, and household size. The model for Tanzania controlled for intervention assignment, age, educational attainment, asset score, household size, and land size.

Figure S17 – Ownership of assets and crop diversity

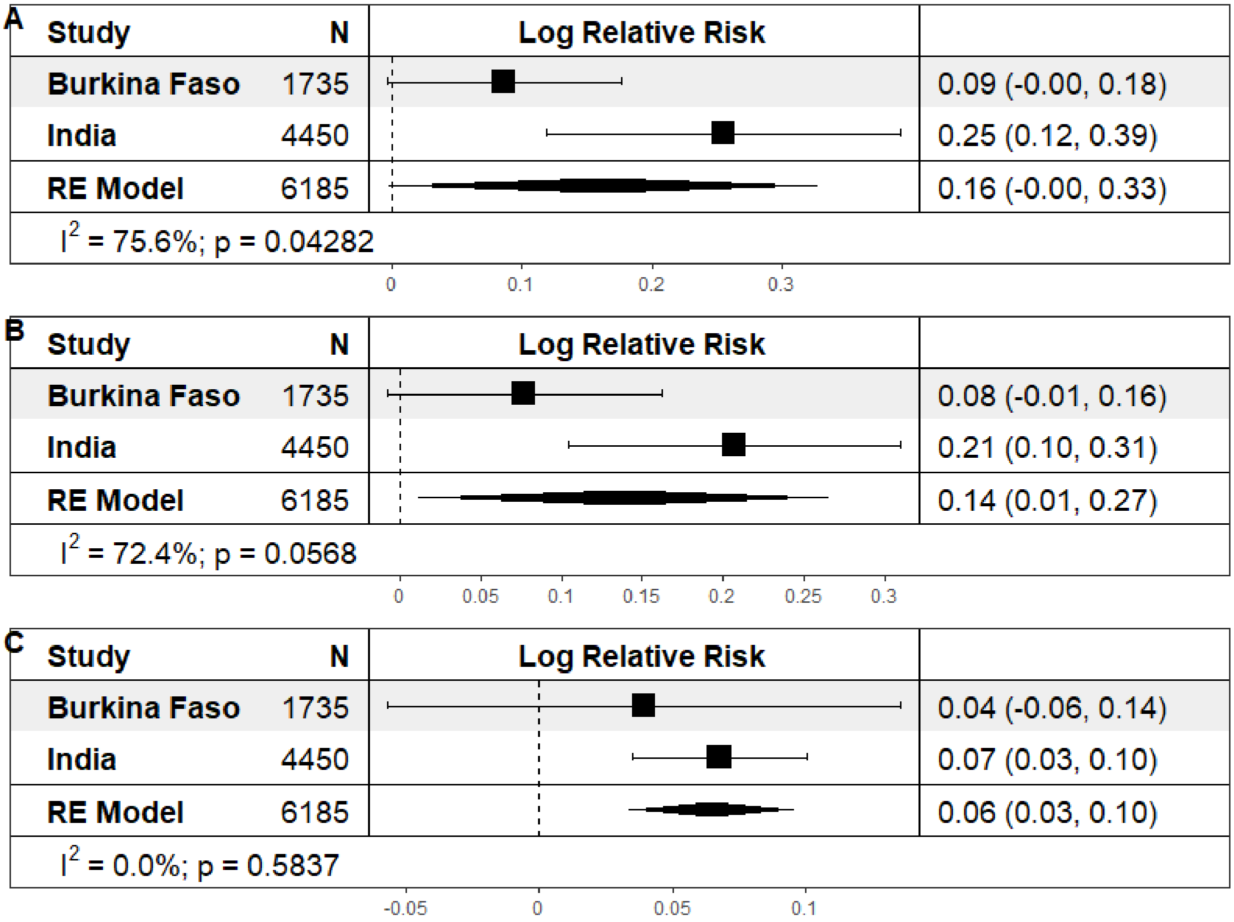

A corresponds to total food crop diversity, **B** corresponds to total food group diversity (range: 0 – 7 food groups), and **C** corresponds to cultivation of nutrient dense crops (defined as such if they reported cultivating at least one crop in the following food group categories: pulses, nuts and seeds, dark green leafy vegetables, other vitamin-A rich fruits and vegetables, other vegetables, or other fruit. Non-cultivators of nutrient dense crops were those who grew only grains, roots, and tubers or if they grew no food groups (meaning not growing anything at all or only growing cash crops).

Ownership of assets was defined as empowered if owned at least one large asset or at least two small assets.

We pooled country-specific adjusted estimates using a random effects model using the DerSimonian and Laird method.

The adjusted model for Burkina Faso controlled for intervention assignment, age, educational attainment, household size, and polygyny. The model for India controlled for intervention assignment, age, educational attainment, household size, and land size. The model for Malawi controlled for intervention assignment, age, educational attainment, and household size. The model for Tanzania controlled for intervention assignment, age, educational attainment, household size, and land size.

Figure S18 – Access to and decisions on credit and crop diversity

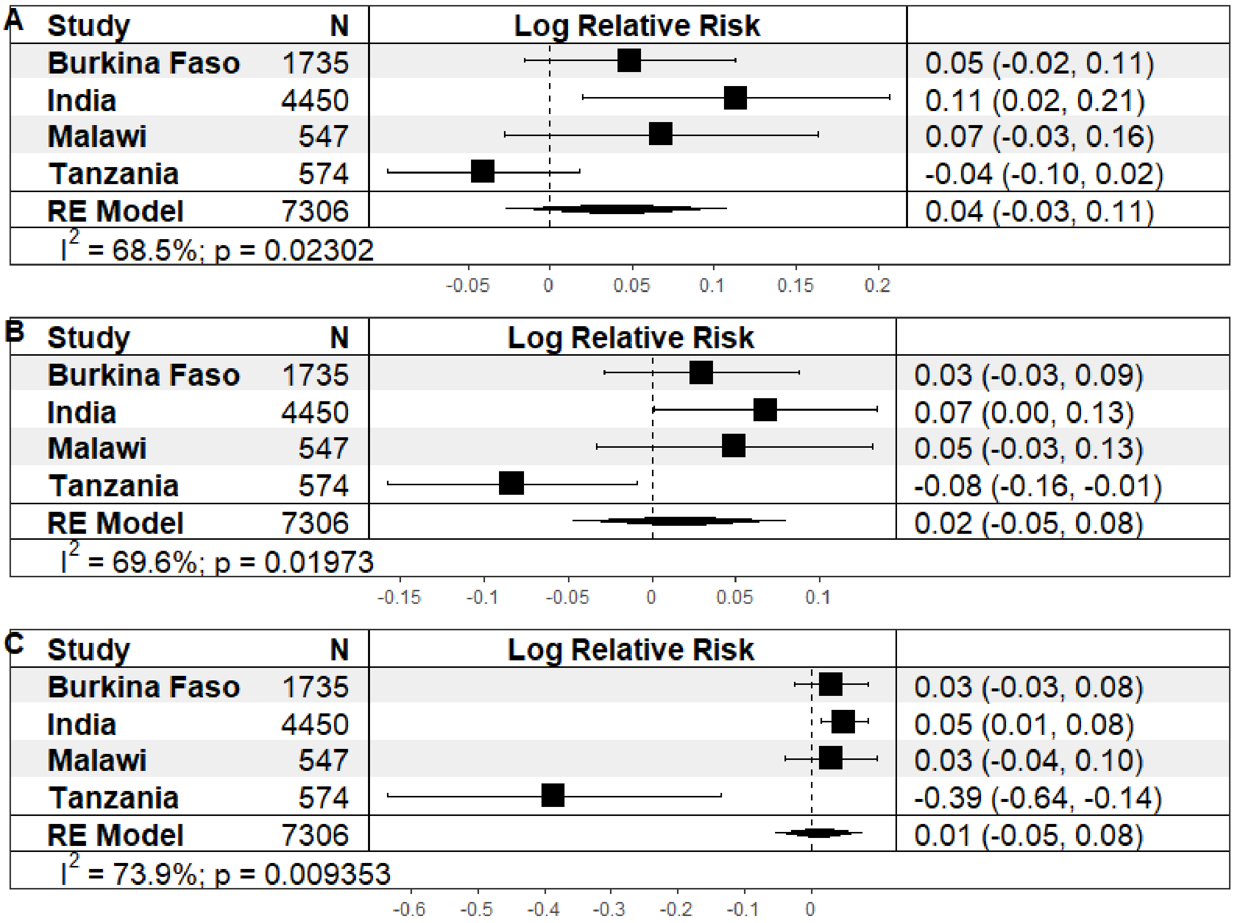

A corresponds to total food crop diversity, **B** corresponds to total food group diversity (range: 0 – 7 food groups), and **C** corresponds to cultivation of nutrient dense crops (defined as such if they reported cultivating at least one crop in the following food group categories: pulses, nuts and seeds, dark green leafy vegetables, other vitamin-A rich fruits and vegetables, other vegetables, or other fruit. Non-cultivators of nutrient dense crops were those who grew only grains, roots, and tubers or if they grew no food groups (meaning not growing anything at all or only growing cash crops).

Access to and decisions on credit was defined as empowered if using at least one source of credit or made decision solely regarding at least one source of credit given that household has access.

We pooled country-specific adjusted estimates using a random effects model using the DerSimonian and Laird method.

The adjusted model for Burkina Faso controlled for intervention assignment, age, educational attainment, asset score, household size, polygyny, and active membership of a credit/microfinance lending group. The model for India controlled for intervention assignment, age, educational attainment, asset score, household size, land size, and active membership of a credit/microfinance lending group. The model for Malawi controlled for intervention assignment, age, educational attainment, asset score, household size, and active membership of a credit/microfinance lending group. The model for Tanzania controlled for intervention assignment, age, educational attainment, asset score, household size, land size, and active membership of a credit/microfinance lending group.

Figure S19 – Group membership and crop diversity

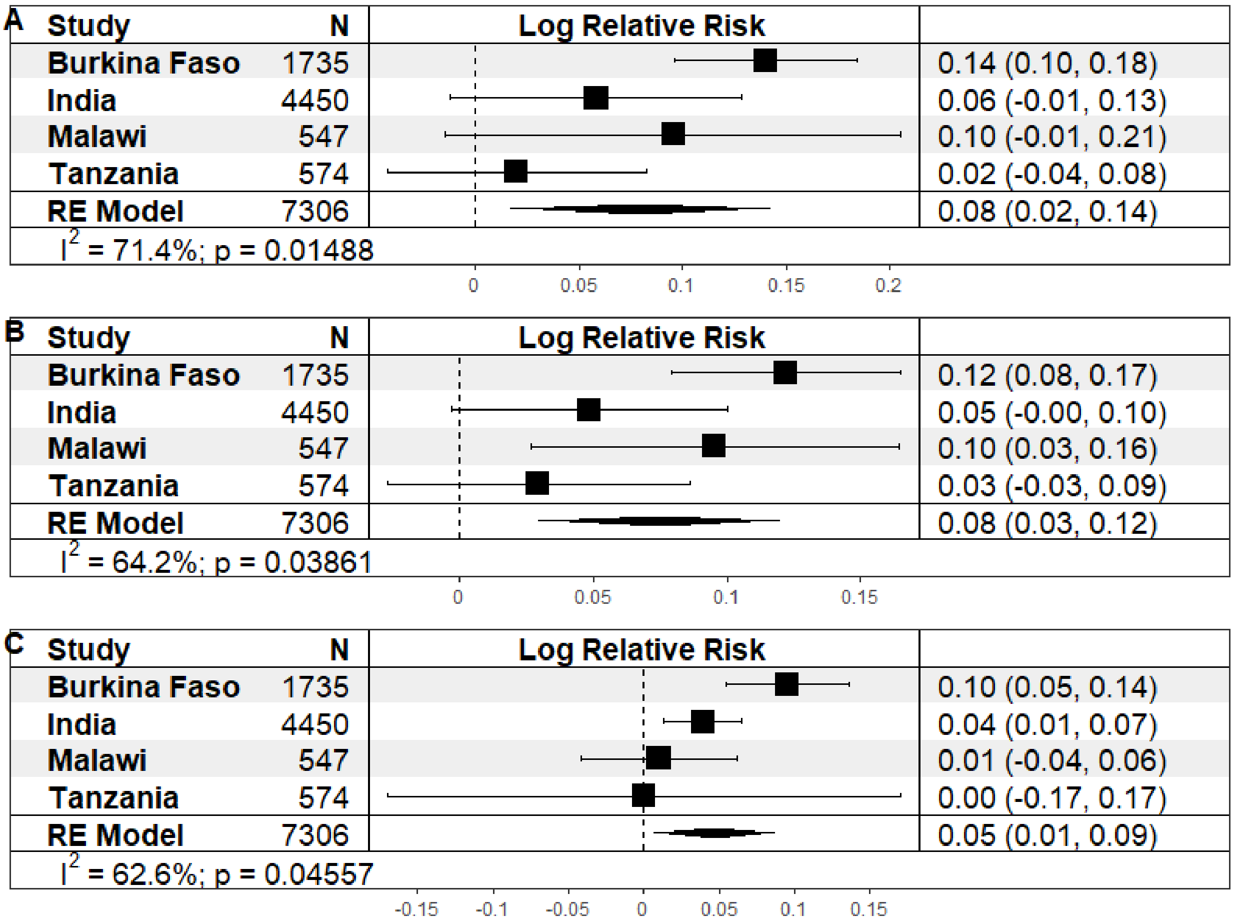

A corresponds to total food crop diversity, **B** corresponds to total food group diversity (range: 0 – 7 food groups), and **C** corresponds to cultivation of nutrient dense crops (defined as such if they reported cultivating at least one crop in the following food group categories: pulses, nuts and seeds, dark green leafy vegetables, other vitamin-A rich fruits and vegetables, other vegetables, or other fruit. Non-cultivators of nutrient dense crops were those who grew only grains, roots, and tubers or if they grew no food groups (meaning not growing anything at all or only growing cash crops).

Group membership was defined as empowered if active member in at least one group.

We pooled country-specific adjusted estimates using a random effects model using the DerSimonian and Laird method.

The adjusted model for Burkina Faso controlled for intervention assignment, age, educational attainment, asset score, household size, and polygyny. The model for India controlled for intervention assignment, age, educational attainment, asset score, household size, and land size. The model for Malawi controlled for intervention assignment, age, educational attainment, asset score, and household size. The model for Tanzania controlled for intervention assignment, age, educational attainment, asset score, household size, and land size.

Figure S20 – Work balance and crop diversity

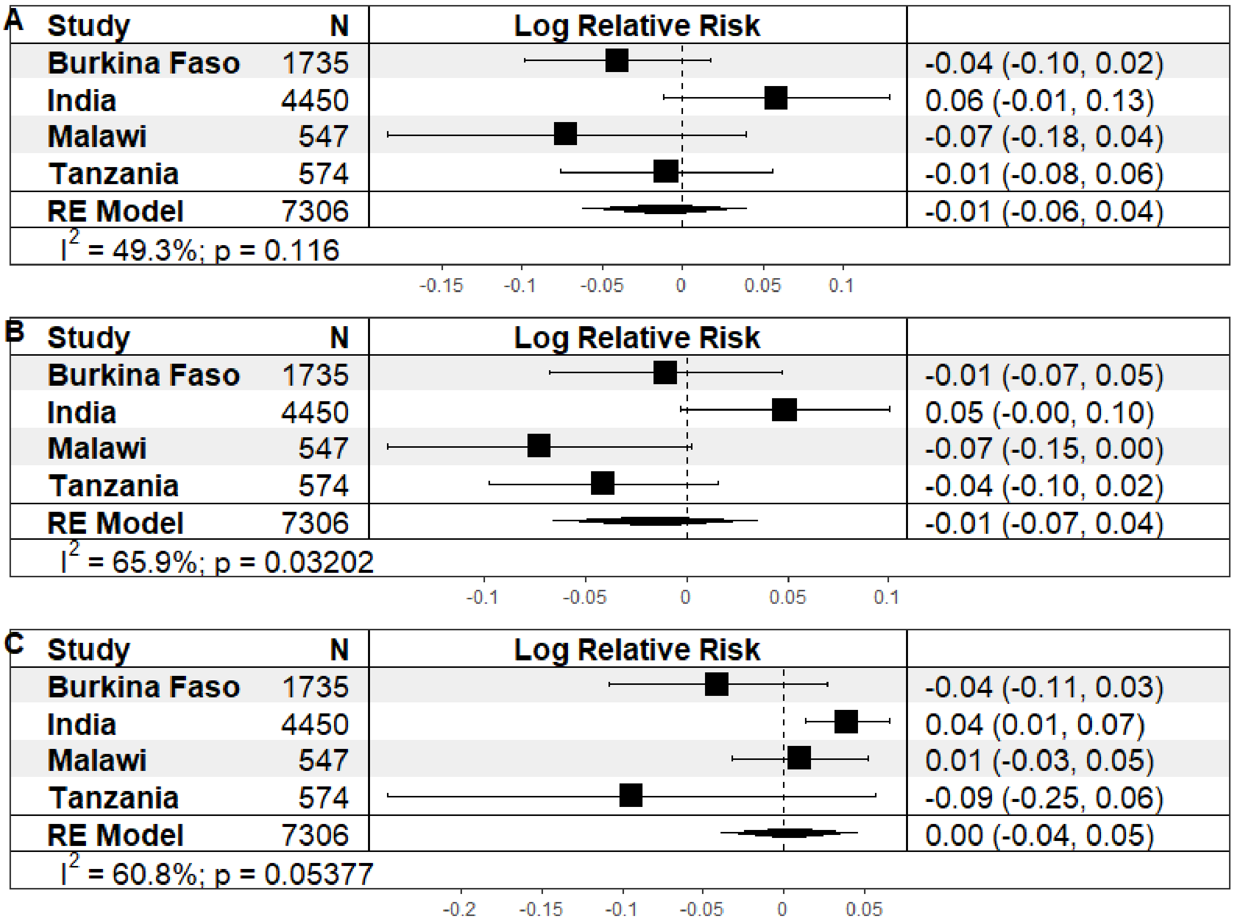

A corresponds to total food crop diversity, **B** corresponds to total food group diversity (range: 0 – 7 food groups), and **C** corresponds to cultivation of nutrient dense crops (defined as such if they reported cultivating at least one crop in the following food group categories: pulses, nuts and seeds, dark green leafy vegetables, other vitamin-A rich fruits and vegetables, other vegetables, or other fruit. Non-cultivators of nutrient dense crops were those who grew only grains, roots, and tubers or if they grew no food groups (meaning not growing anything at all or only growing cash crops).

Work balance was defined as empowered if works less than 10.5 productive hours in one day. This does not consider time dedicated to childcare.

We pooled country-specific adjusted estimates using a random effects model using the DerSimonian and Laird method.

The adjusted model for Burkina Faso controlled for intervention assignment, age, educational attainment, asset score, household size, and polygyny. The model for India controlled for intervention assignment, age, educational attainment, asset score, household size, and land size. The model for Malawi controlled for intervention assignment, age, educational attainment, asset score, and household size. The model for Tanzania controlled for intervention assignment, age, educational attainment, asset score, household size, and land size.

Figure S21 – Productive work hours including childcare and crop diversity

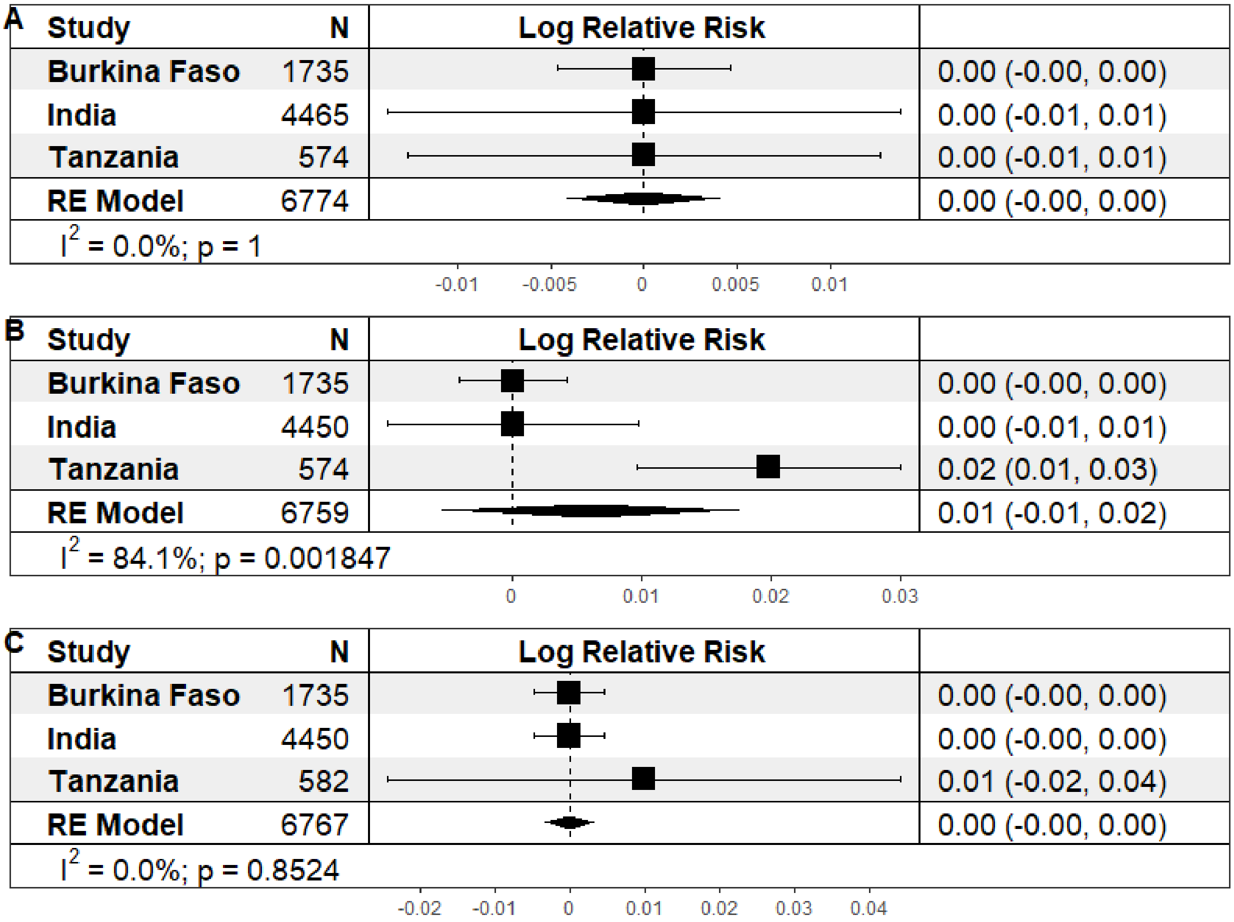

A corresponds to total food crop diversity, **B** corresponds to total food group diversity (range: 0 – 7 food groups), and **C** corresponds to cultivation of nutrient dense crops (defined as such if they reported cultivating at least one crop in the following food group categories: pulses, nuts and seeds, dark green leafy vegetables, other vitamin-A rich fruits and vegetables, other vegetables, or other fruit. Non-cultivators of nutrient dense crops were those who grew only grains, roots, and tubers or if they grew no food groups (meaning not growing anything at all or only growing cash crops).

Productive work hours were defined as the number of hours dedicated to productive work per day per individual, including childcare.

We pooled country-specific adjusted estimates using a random effects model using the DerSimonian and Laird method.

The adjusted model for Burkina Faso controlled for intervention assignment, age, educational attainment, asset score, household size, and polygyny. The model for India controlled for intervention assignment, age, educational attainment, asset score, household size, and land size. The model for Malawi controlled for intervention assignment, age, educational attainment, asset score, and household size. The model for Tanzania controlled for intervention assignment, age, educational attainment, asset score, household size, and land size.

Figure S22 – Work balance including childcare and crop diversity

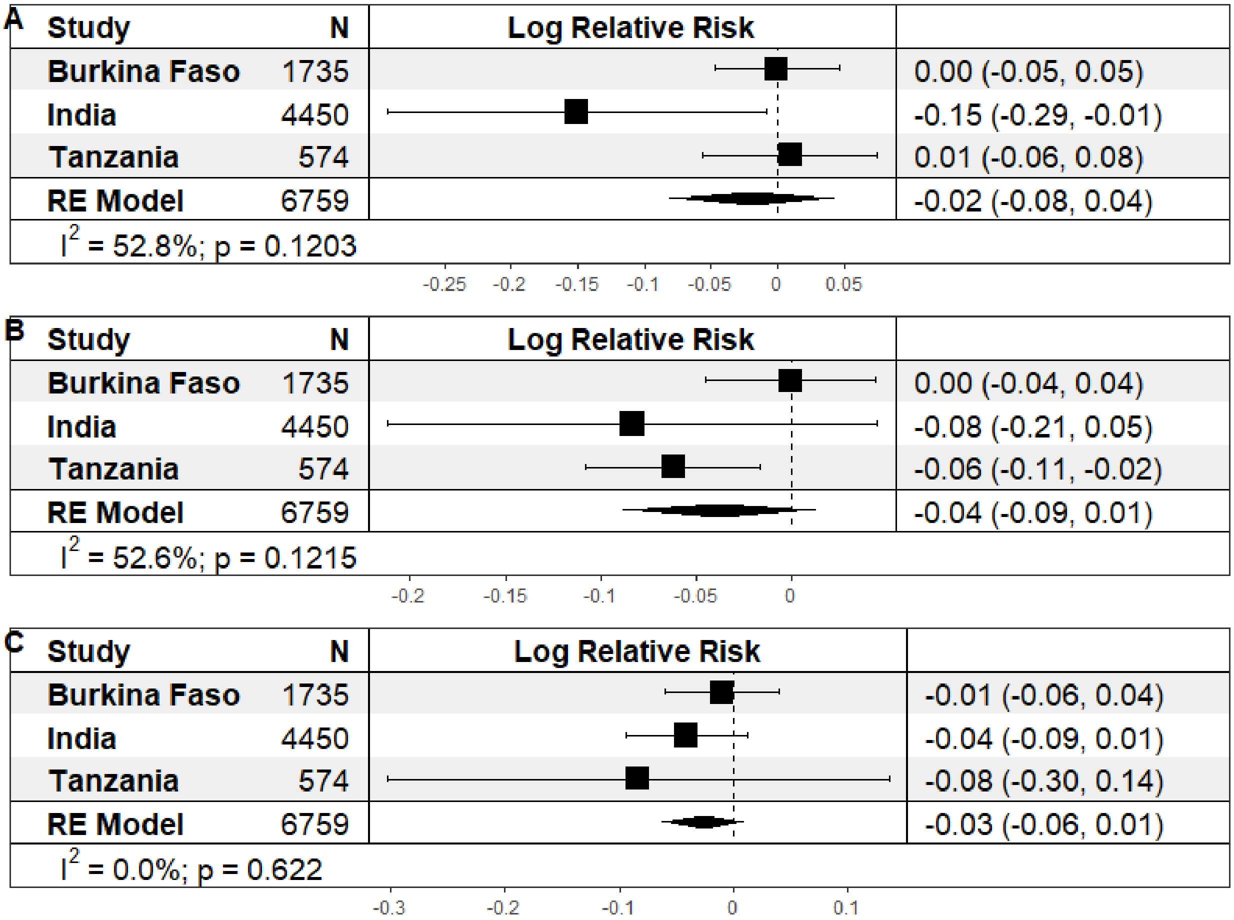

A corresponds to total food crop diversity, **B** corresponds to total food group diversity (range: 0 – 7 food groups), and **C** corresponds to cultivation of nutrient dense crops (defined as such if they reported cultivating at least one crop in the following food group categories: pulses, nuts and seeds, dark green leafy vegetables, other vitamin-A rich fruits and vegetables, other vegetables, or other fruit. Non-cultivators of nutrient dense crops were those who grew only grains, roots, and tubers or if they grew no food groups (meaning not growing anything at all or only growing cash crops).

Work balance was defined as empowered if works less than 10·5 productive hours in one day, including childcare.

We pooled country-specific adjusted estimates using a random effects model using the DerSimonian and Laird method.

The adjusted model for Burkina Faso controlled for intervention assignment, age, educational attainment, asset score, household size, and polygyny. The model for India controlled for intervention assignment, age, educational attainment, asset score, household size, and land size. The model for Malawi controlled for intervention assignment, age, educational attainment, asset score, and household size. The model for Tanzania controlled for intervention assignment, age, educational attainment, asset score, household size, and land size.

Supplementary workbook 1 Women's empowerment and crop diversity in Burkina Faso

- **Table S10 Input into productive decisions and crop diversity in Burkina Faso**
- **Table S11 Input into productive decisions and crop diversity in Burkina Faso**
- **Table S12 Ownership of Assets and Crop Diversity in Burkina Faso**
- **Table S13 Ownership of Assets and Crop Diversity in Burkina Faso**
- **Table S14 Ownership of Agricultural Assets and Crop Diversity in Burkina Faso**
- **Table S15 Access to and decisions about credit and Crop Diversity in Burkina Faso**
- **Table S16 Access to credit and Crop Diversity in Burkina Faso**
- **Table S17 Decisions on credit and Crop Diversity in Burkina Faso**
- **Table S18 Group Membership and Crop Diversity in Burkina Faso**
- **Table S19 Group Membership and Crop Diversity in Burkina Faso**
- **Table S20 Work Balance and Crop Diversity in Burkina Faso**
- **Table S21 Productive Work Hours and Crop Diversity in Burkina Faso**
- **Table S22 Work Balance including childcare and Crop Diversity in Burkina Faso**
- **Table S23 Productive Work Hours including childcare and Crop Diversity in Burkina Faso**

Supplementary workbook 2 Women's empowerment and crop diversity in India

- **Table S24 Input into productive decisions and crop diversity in India**
- **Table S25 Input into productive decisions and crop diversity in India**
- **Table S26 Ownership of Assets and Crop Diversity in India**
- **Table S27 Ownership of Assets and Crop Diversity in India**
- **Table S28 Ownership of Agricultural Assets and Crop Diversity in India**
- **Table S29 Access to and decisions about credit and Crop Diversity in India**
- **Table S30 Access to credit and Crop Diversity in India**
- **Table S31 Decisions on credit and Crop Diversity in India**
- **Table S32 Group Membership and Crop Diversity in India**
- **Table S33 Group Membership and Crop Diversity in India**
- **Table S34 Work Balance and Crop Diversity in India**
- **Table S35 Productive Work Hours and Crop Diversity in India**
- **Table S36 Work Balance including childcare and Crop Diversity in India**
- **Table S37 Productive Work Hours including childcare and Crop Diversity in India**

Supplementary workbook 3 Women's empowerment and crop diversity in Malawi

- **Table S38 Input into productive decisions and crop diversity in Malawi**
- **Table S39 Input into productive decisions and crop diversity in Malawi**
- **Table S40 Access to and decisions about credit and Crop Diversity in Malawi**
- **Table S41 Access to credit and Crop Diversity in Malawi**
- **Table S42 Decisions on credit and Crop Diversity in Malawi**
- **Table S43 Group Membership and Crop Diversity in Malawi**
- **Table S44 Group Membership and Crop Diversity in Malawi**
- **Table S45 Work Balance and Crop Diversity in Malawi**
- **Table S46 Productive Work Hours and Crop Diversity in Malawi**

Supplementary workbook 4 Women's empowerment and crop diversity in Tanzania
Table S47 Input into Productive Decisions and Crop Diversity in Tanzania
Table S48 Input into Productive Decisions and Crop Diversity in Tanzania
Table S49 Access to and decisions on credit and Crop Diversity in Tanzania
Table S50 Access to and decisions on credit and Crop Diversity in Tanzania
Table S51 Group Membership and Crop Diversity in Tanzania
Table S52 Group Membership and Crop Diversity in Tanzania
Table S53 Work balance and crop diversity in Tanzania
Table S54 Productive work hours and Crop Diversity in Tanzania
Table S55 Work balance including childcare and crop diversity in Tanzania
Table S56 Productive work hours including childcare and crop diversity in Tanzania
